# Evidence accumulation during self-referential social processing captures social functioning and psychopathology dimensions in a transdiagnostic sample

**DOI:** 10.1017/S003329172610573X

**Published:** 2026-09-03

**Authors:** Carly A. Lasagna, Kathryn C. Kemp, Laura Locarno, Kelly Mathis, Katharine N. Thakkar, Costanza Colombi, Cynthia Z. Burton, Timothy J. Pleskac, Ivy F. Tso

**Affiliations:** 1 Department of Psychology, University of Michigan, USA; 2 Department of Psychiatry & Behavioral Health, The Ohio State University, USA; 3 School of Social Work, University of Michigan, Ann Arbor, USA; 4 Department of Psychology, Michigan State University, USA; 5 IRCCS Fondazione Stella Maris, Italy; 6 Department of Psychiatry, University of Michigan, USA; 7 Department of Psychological and Brain Sciences, Indiana University Bloomington, USA

**Keywords:** autism, computational modeling, computational psychiatry, drift diffusion model, evidence accumulation, gaze perception, psychosis, social anxiety, social functioning social cognition, transdiagnostic

## Abstract

**Background:**

Psychosis, social anxiety, and autism are clinical constructs that share important phenotypic features, particularly social dysfunction. A critical determinant of social functioning is an individual’s ability to recognize when social cues, such as eye gaze, are meant for them. Emerging computational modeling data in psychosis suggest that altered gaze perception stems from deficits in evidence accumulation – the process of gathering and integrating information – when processing social cues. We investigated whether aberrant evidence accumulation during gaze perception is associated with social functioning or, as a secondary aim, with psychopathology in a transdiagnostic sample with varying levels of psychotic, socially anxious, or autistic characteristics.

**Methods:**

We examined gaze perception in 111 individuals (aged 14–30) with varying levels of social dysfunction, psychosis proneness, social anxiety, and autistic traits. Drift diffusion models (DDM) characterized key processes driving perceptual judgements in a self-referential gaze perception task, including the efficiency of evidence accumulation. We tested whether evidence accumulation was associated with social functioning, social cognition, and these different psychopathology dimensions.

**Results:**

Impaired evidence accumulation during gaze perception showed strong associations with impaired social cognition and modest associations with diminished social functioning and elevated psychosis and social anxiety symptoms. Evidence accumulation was not associated with autism traits.

**Conclusions:**

Evidence accumulation for social cues relates to social cognition – a key determinant of social functioning. It may also be relevant to real-world social functioning and multiple psychopathology dimensions, including psychosis and social anxiety. Therefore, evidence accumulation should be investigated as a mechanism supporting social difficulties and related psychopathology.

## Introduction

Social dysfunction is a key transdiagnostic feature of psychopathology that is associated with diminished quality of life and worsened prognosis (de Lijster et al., 2018; Handest et al., 2023; Hoertnagl et al., 2020). For instance, psychosis spectrum disorders, autism spectrum disorder, and social anxiety are all accompanied by prominent social dysfunction (Blanchard, Mueser, & Bellack, 1998; Bottema-Beutel, Kim, & Crowley, 2019; Davila & Beck, 2002), such as challenges initiating and sustaining meaningful interpersonal relations and navigating interpersonal conflict. Thus, social dysfunction is an important functional domain to target in interventions for psychopathology. Identifying the many factors that lead to impairment in social functioning remains at the forefront of research, in the hope of better tailoring treatments and alleviating the burden of mental illness.

A critical determinant of social functioning is an individual’s ability to recognize when social cues, like a person waving ‘hello’ or calling their name, are meant for them. Among the most crucial is *self-referential gaze perception*: the ability to discern whether others are looking at them (Emery, 2000; Itier & Batty, 2009). Gaze processing underlies more advanced social processes, such as interpreting others’ attention and intention (Shepherd, 2010), and its disruption is associated with poorer functional outcomes (Campbell et al., 2006; Wastler & Lenzenweger, 2018). Unsurprisingly, disruptions in gaze perception are observed across various forms of psychopathology accompanied by social dysfunction. For example, individuals with social anxiety (Chen, Nummenmaa, & Hietanen, 2017; Gamer, Hecht, Seipp, & Hiller, 2011; Harbort et al., 2013; Schulze, Lobmaier, Arnold, & Renneberg, 2013) and psychosis (Chan et al., 2021; Hooker & Park, 2005; Tso, Mui, Taylor, & Deldin, 2012; Tso, Taylor, & Johnson, 2021) often overperceive eye contact. Those with autism (Pantelis & Kennedy, 2017) and psychosis make slower, less accurate, and/or less consistent gaze judgments (Grove et al., 2021; Tso et al., 2021; Zhu et al., 2007). Disrupted gaze processing may thereby provide a transdiagnostic explanation for prominent social dysfunction that occurs across these disorders. Examining these features in a transdiagnostic sample, rather than arbitrarily defined diagnoses, offers the opportunity to identify shared and distinct mechanisms of social dysfunction across diagnostic categories.

**Figure 1.**
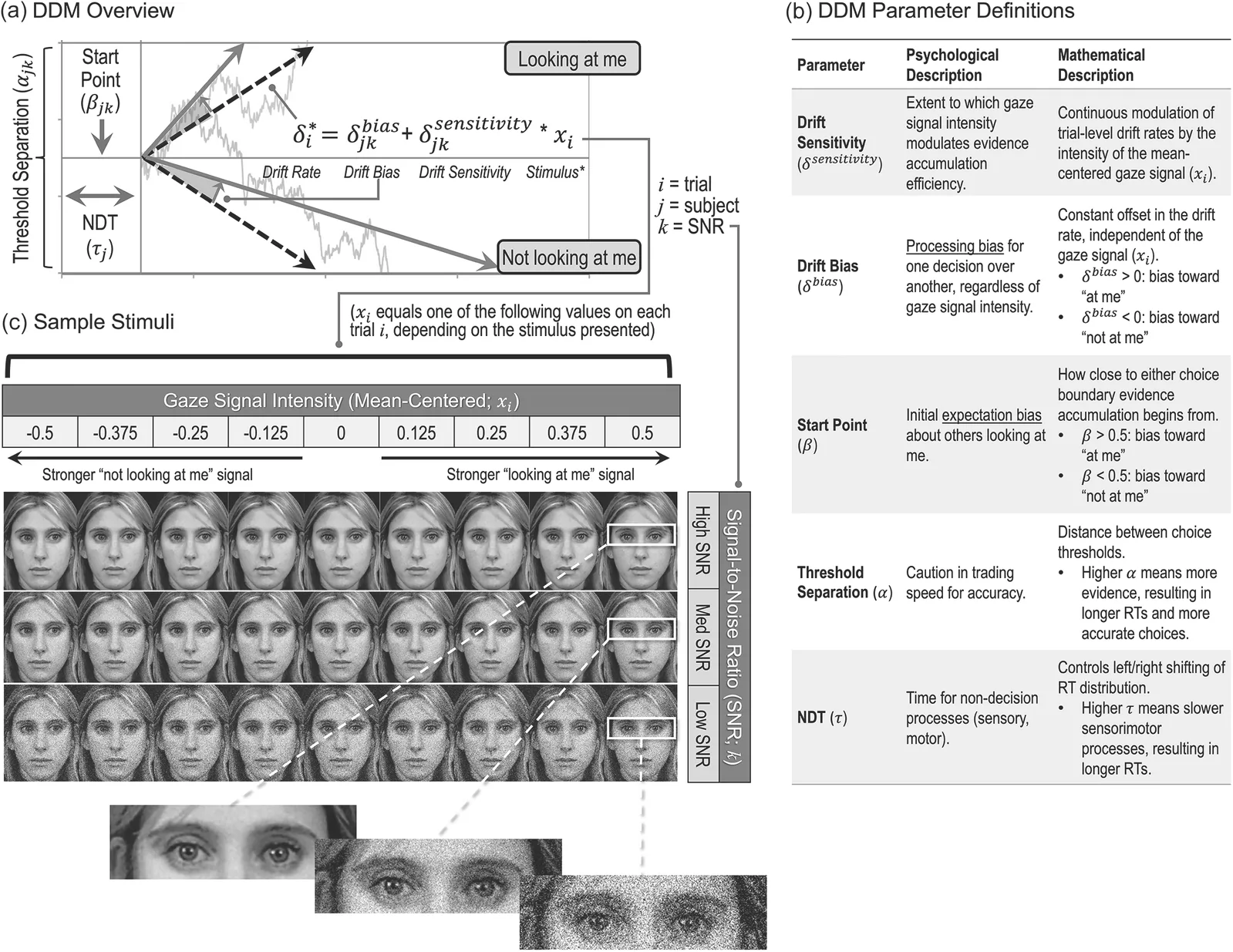
Drift Diffusion Model (DDM) account of self-referential gaze perception. *Note*: According to the DDM, gaze perception can be conceptualized as a process of evidence accumulation: the process of extracting and integrating information from the environment to make a decision. Each subject begins accumulating evidence from a *start point*, indexing initial expectations that others are looking at them. From there, how efficiently they accumulate information is determined by their drift rate. Higher magnitude drift rates mean more efficiency and faster and more accurate/consistent responses. On a given trial, their drift rate is determined by: 1) how strong the physical gaze signal is on a given trial (*x_i_*; which is mean-centered and shown above in red), 2) their *drift sensitivity*, a parameter capturing how sensitive they are to the gaze signal in the physical stimulus, and 3) their *drift bias*, a parameter capturing their perceptual bias for self-referential information, regardless of the actual gaze signal. Here, trial-level drift rates underwent additional transformations that allowed for a non-linear mapping between gaze signals (*x_i_*) and trial-level drift rates (*δ_i_*; see Supplementary Material S3 for details). The *threshold separation* controls how much evidence is needed before deciding, indexing caution in trading speed for accuracy. Finally, *non-decision time* (NDT) captures time for encoding and motor execution. To approximate the multiple layers of ambiguity that individuals encounter in the real world (i.e., naturally ambiguous social cues, often judged under less-than-ideal viewing conditions), this task manipulates both the gaze signal (*x_i_*) of the stimulus (shown above in red) and the overall signal-to-noise ratio (SNR) of faces (*k;* shown above in green). The gaze signal (*x_i_*) varies pseudorandomly from trial-to-trial and is modeled as a continuous predictor of the trial-level drift rate. Alternatively, the SNR manipulation is arranged in three blocks, where block 1 is high SNR (no noise), block 2 is medium SNR (low noise), and block 3 is low SNR (high noise). The effect of SNR is modeled by estimating the drift sensitivity, drift bias, threshold separation, and start point as separate, correlated parameters across the three SNR levels (*k*). Together, these key parameters determine choice and RT behavior on any given trial (*i*) for a given subject (*j*). The time at which the accumulated evidence first hits a boundary (yielding a RT) and the boundary that is hit (yielding a choice) are modeled as a joint outcome of a Wiener diffusion process that is governed by four parameters: (1) trial-level drift rate (determined by subject-level drift sensitivity, subject-level drift bias, and trial-level gaze signal), (2) subject-level threshold separation, (3) subject-level start point, and (4) subject-level NDT. As such, the DDM parameters account for information about RT and choices simultaneously. Complete model specifications are provided in Supplementary Material S3. Figure 1. long description.

Importantly, mechanistic interpretations of putative associations between gaze processing deficits, functioning, and symptoms are complicated by the multiple cognitive processes that underpin self-directed gaze perception. These are difficult to tease apart using traditional performance indices that are aggregated across trials (e.g., mean accuracy and reaction time [RT]). Fortunately, computational models offer a unique approach to quantify those processes using systems of theory-driven mathematical equations. Our recent work (Lasagna, Pleskac, Weigard, & Tso, 2026a; Lasagna, Tso, Blain, & Pleskac, 2026b) showed that the cognitive mechanisms driving gaze perception can be captured using Drift Diffusion Models (DDM; Ratcliff & McKoon, 2008; Figure 1). DDMs describe two-choice decisions as a process by which noisy evidence accumulates over time until it reaches a decision threshold; in this way, it models both choices and RT. In the context of a self-referential gaze perception task, individuals start with an initial expectation and, from there, they begin accumulating evidence about where that person is looking. The quality of evidence accumulated is not fixed but depends on the external stimulus (intensity of the gaze signal, or how clearly self-directed gaze cues are) and the attributes of the individual (how sensitive they are to subtle changes in self-directed gaze). This characteristic of evidence accumulation can be assessed by systematically manipulating the intensity of the gaze signals across experimental trials and then mathematically modeling a “drift sensitivity” parameter that quantifies the extent to which evidence accumulation efficiency is modulated by gaze signal intensity. Psychologically, higher drift sensitivity means that the individual is more attuned to self-directed gaze cues in their environment. For simplicity, we call this “*evidence accumulation sensitivity*” hereafter. Although the DDM outlines several additional cognitive processes that impact decision-making (all described in Figure 1), our recent work in samples with schizophrenia demonstrated that evidence accumulation sensitivity, among all cognitive processes of the DDM during gaze processing, was most strongly related to social cognition (Lasagna, Tso, Blain, & Pleskac, 2026b) and paranoia (Lasagna, Pleskac, Weigard, & Tso, 2026a). As such, the present analysis focuses on this specific process.

However, it remains unclear if evidence accumulation (in)sensitivity to self-directed gaze cues is unique to those with psychosis, or represents a shared mechanism of psychopathologies accompanied by social dysfunction. Emerging evidence suggests that inefficient evidence accumulation across various cognitive tasks may represent a common mechanistic disruption that may confer transdiagnostic risk for various psychopathologies (Sripada & Weigard, 2021; Weigard & Sripada, 2021). It is, therefore, possible that insensitive evidence accumulation observed in schizophrenia during gaze processing could represent a risk factor for not just the disorder (i.e., schizophrenia), but also other forms of psychopathology characterized by significant social dysfunction. Transdiagnostic investigations are needed to test this directly.

### Present study

The present study examines the cognitive processes underlying altered self-referential gaze perception and their clinical and functional relevance in a relatively young, transdiagnostic sample spanning a full spectrum (from none to marked) of social dysfunction. This sample is therefore ideal because: 1) ages 14–30 represent a time of greatest risk for developing psychopathology (Kessler et al., 2007) and social difficulties, and 2) it includes psychopathologies (psychosis proneness, autism, and social anxiety) that are highly comorbid and characterized by prominent social dysfunction. To approximate the multiple layers of ambiguity that individuals encounter when perceiving eye contact in the real world, we manipulated both the “gaze signal” (i.e., gaze direction) and the signal-to-noise ratio (SNR) of faces in the gaze perception task (Figure 1). We hypothesized that, across SNR levels, greater evidence accumulation sensitivity to gaze cues would predict better social cognition (Lasagna, Tso, Blain, & Pleskac, 2026b), and by extension, social functioning. Additionally, we hypothesized that less evidence accumulation sensitivity would predict greater levels of psychosis proneness. Given areas of clinical and cognitive overlap with psychosis, but a lack of cognitive modeling research in these disorders, as a secondary aim we explored the relationships between evidence accumulation sensitivity, autism, and social anxiety traits.

## Methods

Code and outputs are available on GitHub (https://github.com/CarlyLasagna/ddm_socialnoise_transdiagnostic) and OSF (osf.io/57zub). Data will be available upon study completion at nda.nih.gov (Collection ID: 3489).

### Participants and procedure

The sample consisted of 111 participants from a larger study on the mechanisms of social dysfunction (Tso et al., 2020) with complete data from the gaze perception task (described below). Participants had varying levels of psychosis proneness, autism, and social anxiety traits, which are highly comorbid and characterized by prominent social dysfunction. Demographics are provided in Table 1.

**Table 1.** Sample demographics Table 1. long description.

*Domain*	*Variable*	*M*	*SD*	*N*
*Demographics*	Age	23.21	3.96	111
	Sex (% Female)	76%	–	111
	Education (Years)	15.37	2.77	111
	Parent education (Years)	16.50	2.75	105
*Race*	African American (%)	5%	–	111
	Asian (%)	21%	–	111
	Multiracial (%)	4%	–	111
	White (%)	71%	–	111
*Ethnicity*	Hispanic (%)	5%	–	110
*Medications*	Antipsychotic user (%)	13%	–	108
	Antidepressant user (%)	40%	–	108
	Mood stabilizer user (%)	12%	–	108
	Stimulant user (%)	23%	–	108
	Anticholinergic user (%)	4%	–	108
*General intelligence*	IQ	116.58	12.19	111
*Symptoms*	Psychosis proneness – PDI (Total endorsed)	2.83	2.76	106
	Psychosis proneness – PDI (Total distress)	9.05	9.56	98
	Psychosis proneness – CAPS (Total endorsed)	3.75	4.06	106
	Psychosis proneness – CAPS (Total distress)	10.58	11.73	104
	Social anxiety – SADD	1.35	0.95	106
	Social anxiety – SPIN	31.49	17.05	106
	Autism traits – AQ	25.62	9.58	109
	Autism traits – ADOS-social affect	3.55	3.63	110
*Social cognition*	RME (total correct)	28.32	3.77	107
	TASIT (% correct)	80%	8%	111
*Social functioning*	SRS^a^	55.50	31.97	88^b^
	MIRECC GAF-Social^a^	68.64	15.50	105
	GF-Social/Current^a^	6.69	1.63	111
	SASSR-Social^a^	2.44	0.85	104

*Note*: %, proportion; IQ, age/gender corrected full scale intelligence quotient (FSIQ2) on the Wechsler abbreviated scale of intelligence 2nd edition; PDI, Peters et al. delusion inventory; CAPS, Cardiff anomalous perception scale; SADD, Social anxiety disorder dimensional scale; SPIN, Social phobia inventory; AQ, Autism spectrum quotient; ADOS, Autism diagnostic observation schedule 2^nd^ edition; RME, Reading the mind in the eyes test; TASIT, The awareness of social inference test (Short form); SRS, Social responsiveness scale; MIRECC GAF, MIRECC global assessment of functioning; GF, Global functioning scale; SASSR, Social adjustment scale. *N*, participants with complete data.

a Values for social functioning measures are pooled across entire sample, but the breakdown by “dysfunctional” and “functional” subgroups is as follows: SRS, dysfunctional (*M* = 31.75, SD = 22.90) and functional (*M* = 64.40, SD = 30.41), MIRECC GAF-Social, dysfunctional (*M* = 60.55, SD = 10.70) and functional (*M* = 87.09, SD = 5.87), GF-Social/Current, dysfunctional (*M* = 5.87, SD = 1.09) and functional (*M* = 8.72, SD = 0.68), SASSR-Social, dysfunctional (*M* = 2.79, SD = 0.72) and functional (*M* = 1.61, SD = 0.50).

b Higher proportion of data missing than other measures because measures were completed by informants and not all invited informants completed the questionnaire.

Inclusion criteria were as follows: aged 14–30; visual acuity ≥20/30; IQ ≥ 80; and no Mendelian disorder, neurological conditions, or substance use in the last month (see Supplementary Material S1 for additional criteria). To capture a broad range of social functioning, approximately two-thirds of the sample was recruited in the socially “dysfunctional” range (Global Functioning [GF] Scale-Social≤6 -OR- MIRECC Global Assessment of Functioning [GAF]-Social≤69; Piskulic, Addington, Auther, & Cornblatt, 2011, Niv, Cohen, Sullivan, & Young, 2007). The rest were recruited in the socially “functional” range (GF-Social≥7 -OR- MIRECC GAF-Social≥70). Those in the “functional” range had no DSM-5 diagnoses and were not taking psychotropic medications. To ensure sufficient representation of our three clinical dimensions of interest, recruitment aimed for those in the “dysfunctional” range to consist of approximately 20% with a psychotic disorder and 20% with social anxiety disorder (based on Mini International Neuropsychiatric Interview for DSM-5; Sheehan et al., 1998), and 20% with autism spectrum disorder (based on Autism Diagnostic Observation Schedule 2nd Edition [ADOS-2] revised scoring; Hus & Lord, 2014; Lord et al., 2012). Ultimately, in our analytic sample, 47%, 22%, 11%, and 19% respectively, met criteria for social anxiety, autism spectrum, psychosis-spectrum, or multiple disorders.

Participants completed a screening visit (clinical assessments, self-ratings, demographics), an in person visit (eye tracking, gaze perception and social cognitive tasks, observation-based clinical assessments), and an fMRI visit. Data from the current study comes from the first two visits The study was approved by the university’s Institutional Review Board. The authors assert that all procedures contributing to this work comply with the ethical standards of the relevant national and institutional committees on human experimentation and with the Helsinki Declaration of 1975, as revised in 2008. Additional study procedures are detailed in Supplementary Material S1.

### Measures

#### Self-referential gaze perception task

Participants judged whether faces were looking at them (yes/no; Lasagna et al., 2020). Stimuli consisted of grayscale face images of 6 actors (George, Driver, & Dolan, 2001). The task used two manipulations to approximate the multiple layers of ambiguity that individuals encounter when judging eye contact in the real world (Figure 1c). First, the gaze direction was manipulated in gradual increments, spanning 9 “gaze signal” strengths from not looking at me (signal strength = 0) to looking at me (signal strength = 1). Gaze signals varied pseudorandomly from trial to trial. Second, as an added level of ambiguity, the SNR of stimuli was also manipulated by superimposing different levels of Gaussian noise over stimuli. This manipulation reflected the fact that real-world social cues are often judged under less-than-ideal viewing conditions (e.g., judging the facial expression of someone who is wearing a mask or is across the room at a crowded party). SNR manipulations were arranged across three blocks: 1 = high SNR (no noise), 2 = medium SNR (low noise), and 3 = low SNR (high noise). The task was programmed in Experiment Builder (SR Research) and lasted ~15 minutes. It consisted of 324 trials (12 per signal strength × 9 signal strengths × 3 SNR levels), each consisting of a 2000 ms stimulus and a 1-500 ms inter-stimulus interval (ISI). Erroneously fast responses <200 ms were removed (*n* = 6 total trials). In addition to using DDMs, we calculated two more traditional, performance metrics: choice-based sensitivity (derived from a psychometric function fitted to choice behavior that resembled the DDM drift sensitivity parameter (Ratcliff, 2014) and RT. Supplementary Material S2 contains details on how choice-based sensitivity was estimated, additional details about the task/stimuli, and visualizations of basic task behavior, including choice proportions and RT quantiles.

#### Social cognition

Social cognition was assessed using accuracy on the Reading the Mind in the Eyes test (RME; Baron-Cohen et al., 2001) and emotion evaluation and social inference subtests of the Awareness of Social Inference Test-Short Form (TASIT; McDonald, Flanagan, Rollins, & Kinch, 2003). The RME is a perceptual theory-of-mind task consisting of 36 pictures of eyes portraying different emotional states and participants must select the correct emotional state from four options. The TASIT examines emotion evaluation and social inference abilities. The emotion evaluation test includes 10 videos of actors portraying different emotional states. Participants must select the correct emotion from seven options. The social inference test includes 18 videos of social interactions. Participants must answer four yes/no questions about the characters’ actions and mental states for each video. Both tasks allowed unlimited time for responses. For analyses, we extracted a single principal component (variance captured = 45%) of social cognitive ability from *z*-scores of these measures using pcaMethods. This approach was preferred over averaging because it allowed for Bayesian imputation for missing data (Stacklies et al., 2007).

#### Social functioning

Social functioning was assessed using self-report, informant-report, and interviewer ratings. The Social Adjustment Scale-Social Scale-Social/Leisure Scale (SAS-SR-Social) (Weissman, 1999) is a self-report of social functioning. The Social Responsiveness Scale (SRS-2; Constantino & Gruber, 2012) is an informant-report in which an identified individual (e.g., parent, friend) rates the participant’s ability to engage effectively with others. Interviewer-rated measures included the GF-Social/Current (Piskulic, Addington, Auther, & Cornblatt, 2011) and MIRECC GAF-Social (Niv, Cohen, Sullivan, & Young, 2007). For consistency of score interpretation, all measures were recomputed (i.e., z-score*-1 where needed) so that higher scores indicated better social functioning. For analyses, we extracted a single principal component of social functioning (variance captured = 71%) using the approach described above.

#### Symptom dimensions

*Psychosis proneness* was assessed using self-ratings on the Peters Delusion Inventory (PDI; Peters, Joseph, Day, & Garety, 2004) and Cardiff Anomalous Perception Scale (CAPS; Bell, Halligan, & Ellis, 2006). Total items endorsed and distress subscales were used because each provides useful information regarding severity and outcomes (Oh et al., 2024). *Autism traits* were assessed using totals on the self-rated Autism Spectrum Quotient (AQ; Baron-Cohen et al., 2001) and the clinician-rated/observed ADOS-2 (revised scoring; see Supplementary Material S1 for inter-rater reliability; Hus & Lord, 2014; Lord et al., 2012). We focus on the ADOS-2 Social Affect Subscale to capture interaction-based difficulties not captured via the AQ self-report*. Social anxiety* was assessed using self-rated totals on the Social Phobia Inventory (SPIN; Connor et al., 2000) and Social Anxiety Disorder Dimensional Scale (SADD; Lebeau et al., 2012). Higher scores on each measure indicated more severe psychopathology. For each dimension, we extracted a single principal component (variance captured = 47%–90%) using the approach described above. Overlaps between these three dimensions are visualized in Supplementary Material S4.5.

#### Intellectual functioning

General intelligence was assessed using Full-Scale Intelligence Quotient (FSIQ2) scores on the Wechsler Abbreviated Scale of Intelligence, 2nd Edition (Wechsler, 2011).

### Drift diffusion modeling

We extended the DDM from (Lasagna, Pleskac, Weigard, & Tso, 2026a; Figure 1) to capture the cognitive processes contributing to gaze perception across different SNR levels. Complete model specifications and testing details are provided in Supplementary Material S3. To summarize, the gaze perception task approximates multiple layers of ambiguity that individuals encounter when making gaze judgments in the real world (Figure 1) by: (1) continuously manipulating gaze signals (*x_i_*) pseudorandomly from trial-to-trial, *and* (2) categorically manipulating the SNR (*k*) of faces across three task blocks. Continuous effects of gaze signals (*x_i_*) on trial-level drift rates were modeled via the drift sensitivity parameter using the equation in Figure 1. This was done using transformations that allowed for non-linear mappings between gaze signals and drift rates (see Supplementary Material S3). Categorical effects of SNR were modeled by estimating the drift sensitivity, drift bias, threshold separation, and start point as separate, correlated parameters across each SNR level (*k*). As model evaluations for this task and model have been performed previously (Lasagna, Pleskac, Weigard, & Tso, 2026a), we do not repeat those.

Preliminary results indicated that decreasing the SNR reduced drift sensitivity for gaze signals (Supplementary Material S4.1). However, drift sensitivity parameters were *highly* correlated across SNR levels (*r*’s = 0.86–0.98; Supplementary Material S4.2), suggesting that they reflected variants of the same underlying process (Sripada & Weigard, 2021; Stevenson et al., 2024; Weigard & Sripada, 2021). To simplify analyses, we averaged drift sensitivity parameters across SNR levels.

### Statistical analyses

Analyses were performed in RStudio 4.3.1 using Bayesian statistics.

To examine drift sensitivity’s prediction of social cognition, social functioning, psychosis proneness, social anxiety, and autism traits, we employed robust univariate Bayesian regressions in brms using standardized variables (details in Supplementary Material S5). Robust regressions helped mitigate the effects of non-normal distributions (Supplementary Material S4.4). To account for the overrepresentation of females and the broad age range of this sample, a hierarchical approach was used, adding age and sex (Female = 1, Male = 0) at Step 1 and drift sensitivity at Step 2. Previous work shows that associations between functioning, symptoms, and standard measures of social cognition often have small-to-medium effect sizes. While quantifying behavior using cognitive models can strengthen these associations (Haines et al., 2025), it is unlikely that improvements would yield large associations. Therefore, priors for beta coefficients reflected the range of plausible values established in prior work centered at 0 effect (N[0, 0.5]). Supplemental analyses confirmed that effects remained when using weaker priors (N[0,1]) and frequentist statistics (Supplementary Materials S5–S6), suggesting that findings were not merely due to our prior choice. Predictors were evaluated based on: *credibility* (i.e., whether the 95% highest density interval [HDI] for the predictor contained zero); *evidence strength* (i.e., using predictor-level Bayes Factors [BF-Predictor], calculated using Savage-Dickey Density Ratio (Wagenmakers, Lodewyckx, Kuriyal, & Grasman, 2010); and *fit improvements* over an age-and-sex-only model (i.e., using changes in expected log posterior density [ELPD] from leave-one-out cross-validation, and model-level BFs [BF-Model]). Higher ELPD indicates better fit, and ELPD changes within the standard error indicate negligible improvements. BFs index the relative strength of evidence for the alternative (association exists; BF > 1) over the null (no association; BF < 1). Conventional BF interpretations were used (Lee & Wagenmakers, 2014). If BF > 1: 1–3 = anecdotal, 3–10 = moderate,10–30 = strong, 30–100 = very strong, >100 = extreme. If BF < 1: 0.33–1 = anecdotal, 0.10–0.33 = moderate, 0.033–0.10 = strong, 0.01–0.033 = very strong, <0.01 = extreme.

Supplementary Material S6.3–S6.4 including similar exploratory analyses of bias-related DDM parameters (i.e., drift bias and start point). To determine whether results were due to intellectual deficits, or could be observed using behavioral metrics derived from choice or RT data alone, supplemental analyses re-ran regressions covarying for IQ and using RT and choice-based sensitivity as predictors.

## Results

### Associations with functional dimensions

#### Social cognition

Drift sensitivity statistically predicted social cognition (*M* = 0.31 [0.13, 0.50], ΔR^2^ = 8.2%, BF-Predictor = 68.78; Table 2). Relative to an age- and sex-only model, the full model was favored by very strong BF evidence (BF-Model = 36.84) and improved the out-of-sample predictive accuracy of social cognition (ΔELPD = 3.98 > SE = 3.65). This indicates that those with higher drift sensitivity to gaze cues tend to show better social cognitive abilities – a key determinant of social functioning.

**Table 2.** Predicting social functioning and social cognition from drift sensitivity parameters Table 2. long description.

Outcome/ Model/Predictor	Model change^a^			Predictors	
	ELPD (ΔELPD/SE)	BF (Favors/Strength)	R^2^ (ΔR^2^)	M [95% HDI]	BF (Favors/Strength)
*A) Social cognition*					
*Regression 1*	−152.59 (3.98/3.65)	36.84 (Alt/Very Strong)	0.11 (0.08)		
Sex				−0.03 [−0.41, 0.35]	0.40 (Null/Anecdotal)
Age				−0.07 [−0.24, 0.11]	0.23 (Null/Moderate)
Drift sensitivity				**0.31 [0.13, 0.50]^b^**	**68.78 (Alt/Very Strong)**
*B) Social functioning*					
*Regression 2*	−158.79 (2.38/2.32)	3.77 (Alt/Moderate)	0.07 (0.04)		
Sex				−0.30 [−0.71, 0.10]	1.17 (Alt/Anecdotal)
Age				−0.04 [−0.23, 0.14]	0.20 (Null/Moderate)
Drift sensitivity				**0.23 [0.04, 0.42]^b^**	**3.81 (Alt/Moderate)**

*Note: M*, posterior mean of beta coefficient; Alt, alternative hypothesis; HDI, highest density interval; ELPD, expected log posterior density (higher values = better fit); ΔELPD, change relative to reduced age- and sex-only model; SE, standard error.

a Changes relative to reduced age- and sex-only model. Intercepts excluded for brevity but are included in Supplementary Material S5.1–S5.2.

b Credible (bolded for clarity).

#### Social functioning

Drift sensitivity statistically predicted social functioning (*M* = 0.23 [0.04, 0.42], ΔR^2^ = 4.3%; Table 2), supported by moderate BF evidence (BF-Predictor = 3.81). Relative to the reduced age- and sex-only model, the full model was favored by moderate BF evidence (BF-Model = 3.77) and improved the predictive accuracy of social functioning (ΔELPD 2.38 > SE 2.32). This suggests a modest association between higher drift sensitivity to gaze cues and better social functioning.

### Associations with symptom dimensions

#### Psychosis proneness

Drift sensitivity statistically predicted psychosis proneness (*M* = -0.23, [−0.41, −0.02], ΔR^2^ = 4.2%, BF-Predictor = 2.62; Table 3). The full model was favored over the reduced model by anecdotal BF evidence (BF-Model = 2.70) and negligibly improved predictive accuracy of psychosis proneness (ΔELPD 1.82 < SE 2.33). Thus, individuals with diminished evidence accumulation sensitivity to gaze cues experienced more severe psychotic-like experiences, but the association was modest.

**Table 3.** Predicting symptom dimensions from drift sensitivity Table 3. long description.

Outcome/ Model/Predictor	Model change^a^			Predictors	
	ELPD (ΔELPD/SE)	BF (Favors/Strength)	R^2^ (ΔR^2^)	*M* [95% HDI]	BF (Favors/Strength)
*A) Psychosis proneness*					
*Regression 1*	−151.59 (1.82/2.33)	2.70 (Alt/Anecdotal)	0.08 (0.04)		
Sex				0.16 [−0.25, 0.57]	0.55 (Null/Anecdotal)
Age				−0.13 [−0.32, 0.05]	0.48 (Null/Anecdotal)
Drift sensitivity				**−0.23 [−0.41, −0.02]^b^**	**2.62 (Alt/Anecdotal)**
*B) Social anxiety*					
*Regression 2*	−150.28 (1.90/2.14)	2.59 (Alt/Anecdotal)	0.09 (0.04)		
Sex				**0.48 [0.06, 0.88]^b^**	**5.44 (Alt/Moderate)**
Age				−0.09 [−0.28, 0.09]	0.32 (Null/Moderate)
Drift sensitivity				**−0.22 [−0.41, −0.03]^b^**	**2.29 (Alt/Anecdotal)**
*C) Autism traits*					
*Regression 3*	−160.93 (0.37/2.04)	0.88 (Null/Anecdotal)	0.06 (0.03)		
Sex				−0.02 [−0.42, 0.41]	0.44 (Null/Anecdotal)
Age				0.01 [−0.19, 0.19]	0.20 (Null/Moderate)
Drift sensitivity				−0.17 [−0.37, 0.01]	0.91 (Null/Anecdotal)

*Note: M*, posterior mean of beta coefficient; Alt. alternative hypothesis; HDI, highest density interval; ELPD, expected log posterior density (higher values = better fit); ΔELPD, change relative to age- and sex-only reduced model; SE, standard error.

a Changes relative to age- and sex-only reduced model. Intercepts excluded for brevity but included in Supplementary Material S5.3–S5.5.

b Credible (bolded for clarity).

#### Social anxiety

Drift sensitivity statistically predicted social anxiety (*M* = -0.22 [−0.41, −0.03], ΔR^2^ = 3.7%, BF-Predictor = 2.29; Table 3), such that diminished evidence accumulation sensitivity to gaze cues was associated with more prominent social anxiety. This was supported by anecdotal BF evidence (BF-Model = 2.59) and negligible improvements in predictive accuracy (ΔELPD 1.90 < SE 2.14).

#### Autism traits

Drift sensitivity did not statistically predict autism traits (*M* = -0.17 [−0.37, 0.01], ΔR^2^ = 3.1%, BF-Predictor = 0.88; Table 3).

### Supplemental analyses

All results held after controlling for IQ (Supplementary Material S6.1), suggesting they were not merely due to general intellectual deficits.

Past work has placed particular emphasis on links between accuracy on various social cognitive tasks and social functioning. Therefore, post hoc follow-up analyses examined whether drift sensitivity during gaze perception (derived from choice *and* RT data) remained a credible predictor of social functioning after controlling for standard accuracy measures on two popular external social cognition tasks (i.e., RME, TASIT). Results (Supplementary Material S5.2.1) confirmed that drift sensitivity accounted for an additional 4.8% of the variance in social functioning after controlling for age, sex, and accuracy on these external social cognition tasks (*M* = 0.22 [0.02, 0.42]), albeit weakly so (BF-Predictor = 1.73).

Finally, no credible associations with functional or symptom dimensions were found when choice-based sensitivity (derived from a psychometric fitted to only choice data) or RT during gaze perception were used as predictors (Supplementary Material S6.2). Together with earlier results, this suggests that measuring the *efficiency* of social information processing using drift sensitivity (which leverages RT *and* choice data) (Ratcliff, 2014, rather than social cognitive measures based on choice or RT data alone, may strengthen associations with clinical measures and social functioning.

## Discussion

This study examined evidence accumulation during a critical social process – self-referential gaze detection – in a transdiagnostic sample with varying levels of social dysfunction. We found that diminished evidence accumulation sensitivity to self-referential gaze signals was associated with poorer social functioning, worse social cognition, and symptom elevations in multiple psychopathology domains. However, associations with functioning and symptom domains were more modest. These findings may shed light on the difficulties that characterize psychopathologies marked by social dysfunction.

As hypothesized, when evidence accumulation was more attuned to available gaze signals, individuals tended to show better performance on external measures of social cognition and better social functioning across self-reports, informant reports, and clinician ratings. Foremost, these results provide compelling evidence that this computationally defined process relates to more complex social cognitive abilities, like theory-of-mind and social inference. Although associations with functioning were weaker, they provide some indication that the sensitivity of evidence accumulation to gaze cues – a measure derived from choices and response speed – accounted for social functioning beyond choice- or RT-based performance metrics on the gaze perception task or external tests of social cognitive accuracy. One explanation is that social interactions require both fast and accurate processing of social cues, like gaze. For example, during face-to-face conversations, periods of mutual gaze last ~360 ms (Rogers, Speelman, Guidetti, & Longmuir, 2018), providing brief windows to accumulate information. If processing efficiency is poor, tradeoffs may be necessary. For instance, if long viewing times are required to maintain accuracy, others may perceive staring and avoid the viewer (Ellsworth, Carlsmith, & Henson, 1972). At brief viewing times, one settles for less information, often resulting in inaccurate judgments that can impair social interaction (Hamilton, 2016; Sergi & Green, 2003). Therefore, a complete account of the mechanisms of altered gaze perception must jointly characterize the speed and accuracy of perception, but this cannot be done with accuracy or RT metrics alone (Ratcliff, 2014. Instead, social cognitive measures that jointly account for choices and response speed – like the parameters of the DDM – may better capture the abilities that social contexts demand.

Individuals with lower evidence accumulation sensitivity to gaze cues experienced more prominent psychotic-like experiences and social anxiety. This aligns with previous findings in schizophrenia (Lasagna, Pleskac, Weigard, & Tso, 2026a; Lasagna, Tso, Blain, & Pleskac, 2026b) and shows that the sensitivity of evidence accumulation to gaze cues also captures broader transdiagnostic expression of socially relevant psychopathology, notably symptom dimensions marked by social fears. Psychosis and social anxiety show considerable phenomenological overlap (Chau, So, & Barkus, 2023; Horton, Barrantes-Vidal, Silvia, & Kwapil, 2014; Michail & Birchwood, 2009; Stopa, Denton, Wingfield, & Taylor, 2013). Suspiciousness or paranoia in psychosis and fears of scrutiny in social anxiety both entail threat anticipation in social contexts (Freeman et al., 2002). At higher levels of psychosis or social anxiety, individuals may be “hypervigilant” for social information signaling threat or negative evaluation. Such high arousal states are cognitively taxing and may detract from resources available for ongoing information processing. However, it has traditionally been challenging to identify explicit cognitive mechanisms that underlie relationships between social anxiety and psychosis. Critically, our findings propose a common cognitive mechanism that cuts across these symptom dimensions and helps to account for previous findings, including: slower and less precise gaze perception in psychosis (Grove et al., 2021; Tso et al., 2021; Zhu et al., 2007), and less precise gaze perception in social anxiety (Chen, Nummenmaa, & Hietanen, 2017; Gamer, Hecht, Seipp, & Hiller, 2011; Schulze, Lobmaier, Arnold, & Renneberg, 2013). However, our data were cross-sectional and future longitudinal studies are needed to clarify whether insensitivity to social cues is a precursor or a consequence of psychopathology.

While heightened social fears are also common in autism (Montaser et al., 2023), additional features of the autism phenotype may contribute to the lack of association between autism traits and evidence accumulation sensitivity. People with autism often perform well on tasks that involve attending to high-contrast stimuli and local features (Robertson & Baron-Cohen, 2017). Deficits are more likely when higher-level inferencing is required, like inferring another’s intentions from the direction they are looking (Robertson & Baron-Cohen, 2017; Shepherd, 2010). The gaze perception task used here required attending to a single, relatively high-contrast facial feature (i.e., eyes). Consequently, null associations between sensitivity of evidence accumulation during gaze perception and autism traits may be because this task was well-suited to the perceptual strengths of individuals with autism traits. Future studies should examine whether evidence accumulation is disrupted in autism when performing social cognitive tasks that require global information processing, like emotion identification and theory of mind.

From a methodological perspective, insensitive evidence accumulation for social cues – which drives choices and RT – may help reconcile paradoxical or null findings found using accuracy-based social cognitive metrics when potential tradeoffs with response speed were not accounted for (e.g., by not analyzing RT explicitly or by allowing unlimited stimulus viewing time) (Gamer, Hecht, Seipp, & Hiller, 2011; Gu et al., 2024; Harbort, Spiegel, Witthöft, & Hecht, 2017). If the primary mechanism driving dysfunctions stems from diminished evidence accumulation sensitivity, compensatory strategies (e.g., slowing down to “be sure”) may be employed if the task design permits, to improve accuracy/choice consistency at the expense of slower reaction time. As a result, social cognitive performance can appear “intact” or even “improved” if only accuracy (and not RT) is considered, offering only a partial view of decision-making. For example, our previous work found that higher social anxiety was associated with more consistent choices during gaze perception (Gu et al., 2024). However, viewing time was unlimited, and RT was not analyzed. It is possible that diminished evidence accumulation sensitivity combined with an increased caution in those with higher social anxiety was at play. If RT had been accounted for, those results may be consistent with our findings showing the opposite effect. This underscores that routinely investigating speed and accuracy (including tradeoffs between them) is critical to achieving a more complete understanding of social cognitive decision-making in studies of psychopathology.

These findings should be considered in light of several limitations. *First*, we examined evidence accumulation during a basic form of social cue detection, but this represents just one facet of social cognition. However, this same process may also play a role in more complex social cognition, like emotion recognition and theory of mind. To test this, studies could jointly model if a common evidence accumulation process influences decisions across multiple social cognitive tasks at the same time (Stevenson et al., 2024), including simple and more complex ones. Importantly, this should be done using tasks with strong psychometric properties (e.g., Penn Emotion Recognition Task, Hinting Task, our gaze perception task) because the RME and TASIT, used to test convergent validity here, have relatively weak psychometric properties (Higgins, Kaplan, Deschrijver, & Ross, 2024; Lasagna et al., 2020; Pinkham, Harvey, & Penn, 2018). *Second*, general evidence accumulation disruptions across a range of non-social cognitive tasks (e.g., attention, working memory) confer risk for psychopathology (Sripada & Weigard, 2021; Weigard & Sripada, 2021). Although we accounted for intellectual functioning, future studies should model social and non-social tasks within the same participants to clarify whether dysfunctions are specific to social versus general cognitive disruptions, which may differentially relate to clinical phenomena (Blain et al., 2025; Lasagna, Pleskac, Weigard, & Tso, 2026a). *Third*, autism traits were measured using interview- and self-ratings, while social anxiety and psychosis traits were measured using only self-ratings. To tease apart the possible contributions of shared methods variance, future studies should utilize interviewer- and self-rated measures for all three clinical dimensions.

Ultimately, evidence accumulation sensitivity to subtle gaze cues may constitute a common mechanism underlying social dysfunctions observed across psychiatric populations – showing strong associations with determinants of social functioning, and modest associations with levels of real-world social dysfunction. Moreover, this mechanism may be particularly relevant to those psychopathologies characterized by heightened social fears and suspicions, like psychosis and social anxiety – future work should investigate this further. Most importantly, this work supports the notion that intact social functioning requires rapid and accurate processing of social cues, yet most studies of social cognition emphasize accuracy alone. To reflect this, it may be necessary to reconceptualize the measurement of social cognition in a way that accounts for speed and accuracy simultaneously.

## Supporting information

10.1017/S003329172610573X.sm001 Lasagna et al. supplementary material Lasagna et al. supplementary material

## Supplementary material

The supplementary material for this article can be found at http://doi.org/10.1017/S003329172610573X.

## Long descriptions

### Figure 1. Long description

Panel A, D D M Overview, shows a diffusion plot where the y-axis represents evidence accumulation between two boundaries: Looking at me at the top and Not looking at me at the bottom. The x-axis represents time. A vertical bracket on the left indicates Threshold Separation alpha sub j k. A central horizontal line marks the Start Point beta sub j k. To the left of the y-axis, a double-headed arrow indicates N D T tau sub j. Within the plot, jagged lines represent evidence accumulation over time, with arrows indicating drift rates. An equation states delta super asterisk sub i equals delta super bias sub j k plus delta super sensitivity sub j k times x sub i.

Panel B, D D M Parameter Definitions, is a table with three columns: Parameter, Psychological Description, and Mathematical Description.

- Drift Sensitivity delta sensitivity: Extent to which gaze signal intensity modulates evidence accumulation efficiency.

- Drift Bias delta bias: Processing bias for one decision over another; delta super bias greater than 0 favors at me.

- Start Point beta: Initial expectation bias; beta greater than 0.5 favors at me.

- Threshold Separation alpha: Caution in trading speed for accuracy; higher alpha means more evidence needed.

- N D T tau: Time for non-decision processes like sensory and motor execution.

Panel C, Sample Stimuli, displays a horizontal scale of Gaze Signal Intensity x sub i ranging from -0.5 to 0.5. Below this is a grid of 27 face images arranged in three rows by nine columns. The rows are labeled by Signal-to-Noise Ratio S N R k: High S N R (no noise), Med S N R (low noise), and Low S N R (high noise). The columns show a transition from stronger not looking at me signals on the left to stronger looking at me signals on the right. Three zoomed-in crops at the bottom highlight the increasing graininess from High to Low S N R.

Navigate back to Figure 1..

### Table 1. Long description

The table is organized into five columns: Domain, Variable, M (Mean), S D (Standard Deviation), and N (Sample Size).

* Demographics: Age (M 23.21, S D 3.96, N 111), Sex (76% Female, N 111), Education (M 15.37, S D 2.77, N 111), and Parent Education (M 16.50, S D 2.75, N 105).

* Race: White (71%), Asian (21%), African American (5%), and Multiracial (4%), all with N 111.

* Ethnicity: Hispanic (5%, N 110).

* Medications: Antidepressant user (40%), Stimulant user (23%), Antipsychotic user (13%), Mood stabilizer user (12%), and Anticholinergic user (4%), all with N 108.

* General Intelligence: I Q (M 116.58, S D 12.19, N 111).

* Symptoms: Psychosis proneness measured by P D I (Total endorsed M 2.83, Total distress M 9.05) and C A P S (Total endorsed M 3.75, Total distress M 10.58). Social anxiety measured by S A D D (M 1.35) and S P I N (M 31.49). Autism traits measured by A Q (M 25.62) and A D O S-social affect (M 3.55).

* Social Cognition: R M E total correct (M 28.32) and T A S I T percent correct (80%).

* Social Functioning: S R S (M 55.50, N 88), M I R E C C G A F-Social (M 68.64, N 105), G F-Social/Current (M 6.69, N 111), and S A S S R-Social (M 2.44, N 104).

Navigate back to Table 1..

### Table 2. Long description

The table is divided into two main sections: A) Social cognition and B) Social functioning.

Header columns include:

1. Outcome/Model/Predictor.

2. Model change: E L P D (Delta E L P D / S E).

3. Model change: B F (Favors/Strength).

4. Model change: R super 2 (Delta R super 2).

5. Predictors: M [95% H D I].

6. Predictors: B F (Favors/Strength).

Section A: Social cognition (Regression 1).

* Model change: E L P D is minus 152.59 (3.98/3.65); B F is 36.84 (Alt/Very Strong); R super 2 is 0.11 (0.08).

* Predictor Sex: M [95% H D I] is minus 0.03 [minus 0.41, 0.35]; B F is 0.40 (Null/Anecdotal).

* Predictor Age: M [95% H D I] is minus 0.07 [minus 0.24, 0.11]; B F is 0.23 (Null/Moderate).

* Predictor Drift sensitivity: M [95% H D I] is 0.31 [0.13, 0.50]; B F is 68.78 (Alt/Very Strong).

Section B: Social functioning (Regression 2).

* Model change: E L P D is minus 158.79 (2.38/2.32); B F is 3.77 (Alt/Moderate); R super 2 is 0.07 (0.04).

* Predictor Sex: M [95% H D I] is minus 0.30 [minus 0.71, 0.10]; B F is 1.17 (Alt/Anecdotal).

* Predictor Age: M [95% H D I] is minus 0.04 [minus 0.23, 0.14]; B F is 0.20 (Null/Moderate).

* Predictor Drift sensitivity: M [95% H D I] is 0.23 [0.04, 0.42]; B F is 3.81 (Alt/Moderate).

Navigate back to Table 2..

### Table 3. Long description

The table is divided into three sections: A) Psychosis proneness, B) Social anxiety, and C) Autism traits.

Section A: Psychosis proneness (Regression 1)

* Model change: E L P D is minus 151.59 (1.82/2.33); B F is 2.70 (Alt/Anecdotal); R super 2 is 0.08 (0.04).

* Predictors: Sex M is 0.16 [minus 0.25, 0.57], B F 0.55 (Null/Anecdotal). Age M is minus 0.13 [minus 0.32, 0.05], B F 0.48 (Null/Anecdotal). Drift sensitivity M is minus 0.23 [minus 0.41, minus 0.02], B F 2.62 (Alt/Anecdotal).

Section B: Social anxiety (Regression 2)

* Model change: E L P D is minus 150.28 (1.90/2.14); B F is 2.59 (Alt/Anecdotal); R super 2 is 0.09 (0.04).

* Predictors: Sex M is 0.48 [0.06, 0.88], B F 5.44 (Alt/Moderate). Age M is minus 0.09 [minus 0.28, 0.09], B F 0.32 (Null/Moderate). Drift sensitivity M is minus 0.22 [minus 0.41, minus 0.03], B F 2.29 (Alt/Anecdotal).

Section C: Autism traits (Regression 3)

* Model change: E L P D is minus 160.93 (0.37/2.04); B F is 0.88 (Null/Anecdotal); R super 2 is 0.06 (0.03).

* Predictors: Sex M is minus 0.02 [minus 0.42, 0.41], B F 0.44 (Null/Anecdotal). Age M is 0.01 [minus 0.19, 0.19], B F 0.20 (Null/Moderate). Drift sensitivity M is minus 0.17 [minus 0.37, 0.01], B F 0.91 (Null/Anecdotal).

Navigate back to Table 3..
