# Supplementary material for: Evidence accumulation during self-referential social processing captures social functioning and psychopathology dimensions in a transdiagnostic sample
Source: Psychol Med. 2026 Sep 3;56:e261. doi: 10.1017/S003329172610573X (PMC13735847; doi:10.1017/S003329172610573X)
Supplement: Supplementary file 1 [file S003329172610573Xsup001.docx]

Supplement for Lasagna & Kemp et al. (2026)

“Evidence Accumulation During Self-Referential Social Processing Captures Social Functioning and Psychopathology Dimensions in a Transdiagnostic Sample”

*Lasagna CA, Kemp KC, Locarno L, Mathis K, Thakkar KN, Colombi C,
Burton CZ, Pleskac TJ, Tso IF*

**Code and outputs for modeling and analyses are available on OSF and GitHub**

osf.io/57zub

[github.com/CarlyLasagna/ddm_socialnoise_transdiagnostic](https://github.com/CarlyLasagna/ddm_socialnoise_transdiagnostic)

**Data will be available upon termination of the study on the NIH Data Archive**

Collection ID: 3489

[nda.nih.gov/edit_collection.html?id=3489](https://nda.nih.gov/edit_collection.html?id=3489)

**Table of Contents**

[**METHODS SUPPLEMENT	3**](#_Toc235257828)

[1	Participants and Procedures	3](#_Toc235257829)

[2	Gaze Perception Task	5](#_Toc235257830)

[3	Drift Diffusion Model (DDM)	6](#_Toc235257831)

[**ANALYSIS SUPPLEMENT	9**](#_Toc235257832)

[4	Preliminary Analyses	9](#_Toc235257833)

[4.1	SNR Effects	9](#_Toc235257834)

[4.2	Correlations Between Parameters Across SNR Levels	9](#_Toc235257835)

[4.3	Correlations Between Principal Components for Different Clinical Dimensions	10](#_Toc235257836)

[4.4	Distributions of Key Variables	11](#_Toc235257837)

[4.5	Distributions of Clinical Variables by Diagnostic Group	11](#_Toc235257838)

[5	Primary Analyses	15](#_Toc235257839)

[5.1	Predicting Social Cognition (Controlling for Sex, Age)	15](#_Toc235257840)

[5.2	Predicting Social Functioning (Controlling for Sex, Age)	15](#_Toc235257841)

[5.2.1	Predicting Social Functioning (Controlling for Sex, Age, Social Cognition)	15](#_Toc235257842)

[5.3	Predicting Psychosis (Controlling for Sex, Age)	16](#_Toc235257843)

[5.4	Predicting Social Anxiety (Controlling for Sex, Age)	16](#_Toc235257844)

[5.5	Predicting Autism Traits (Controlling for Sex, Age)	16](#_Toc235257845)

[6	Supplemental Analyses	17](#_Toc235257846)

[6.1	Controlling for IQ	17](#_Toc235257847)

[6.1.1	Predicting Social Cognition (Controlling for Sex, Age, IQ)	17](#_Toc235257848)

[6.1.2	Predicting Social Functioning (Controlling for Sex, Age, IQ)	17](#_Toc235257849)

[6.1.2.1	Follow-Up Predicting Social Functioning (Controlling for Sex, IQ, Social Cognition)	17](#_Toc235257850)

[6.1.3	Predicting Psychosis (Controlling for Sex, Age, IQ)	18](#_Toc235257851)

[6.1.4	Predicting Social Anxiety (Controlling for Sex, Age, IQ)	18](#_Toc235257852)

[6.1.5	Predicting Autism Traits (Controlling for Sex, Age, IQ)	18](#_Toc235257853)

[6.2	Traditional Metrics (RT and Choice-Based Sensitivity)	19](#_Toc235257854)

[6.2.1	Predicting Social Cognition (Using RT and Choice-Based Sensitivity Metrics)	19](#_Toc235257855)

[6.2.2	Predicting Social Functioning (Using RT and Choice-Based Sensitivity Metrics)	19](#_Toc235257856)

[6.2.3	Predicting Psychosis (Using RT and Choice-Based Sensitivity Metrics)	19](#_Toc235257857)

[6.2.4	Predicting Social Anxiety (Using RT and Choice-Based Sensitivity Metrics)	20](#_Toc235257858)

[6.2.5	Predicting Autism Traits (Using RT and Choice-Based Sensitivity Metrics)	20](#_Toc235257859)

[6.3	Expectancy Bias (DDM Start Point)	21](#_Toc235257860)

[6.3.1	Predicting Social Cognition (Using DDM Start Point)	21](#_Toc235257861)

[6.3.2	Predicting Social Functioning (Using DDM Start Point)	21](#_Toc235257862)

[6.3.3	Predicting Psychosis (Using DDM Start Point)	21](#_Toc235257863)

[6.3.4	Predicting Social Anxiety (Using DDM Start Point)	22](#_Toc235257864)

[6.3.5	Predicting Autism Traits (Using DDM Start Point)	22](#_Toc235257865)

[6.4	Processing Bias (DDM Drift Bias)	22](#_Toc235257866)

[6.4.1	Predicting Social Cognition (Using DDM Drift Bias)	22](#_Toc235257867)

[6.4.2	Predicting Social Functioning (Using DDM Drift Bias)	22](#_Toc235257868)

[6.4.3	Predicting Psychosis (Using DDM Drift Bias)	23](#_Toc235257869)

[6.4.4	Predicting Social Anxiety (Using DDM Drift Bias)	23](#_Toc235257870)

[6.4.5	Predicting Autism Traits (Using DDM Drift Bias)	23](#_Toc235257871)

[7	References	24](#_Toc235257872)

# METHODS SUPPLEMENT

# 1 Participants and Procedures

The sample came from a larger transdiagnostic study on the mechanisms of social dysfunction, wherein symptom dimensions marked by prominent social difficulties were well-represented, including psychosis proneness, autism traits, and social anxiety. The subset examined in the present study (N = 111) had complete behavioral data from the primary experimental task.

**Inclusion Criteria.** All met study inclusion criteria: aged 14-30; visual acuity ≥ 20/30; IQ ≥ 80 (on Wechsler Abbreviated Scale of Intelligence 2nd Edition age/gender corrected full-scale IQ, based on vocabulary and matrix reasoning subtests; FSIQ2 (Wechsler, 2018)); and no Mendelian disorder, neurological conditions, substance use in last 30 days, or dyslexia. Participants were recruited across the full spectrum of social functioning, where ‘social dysfunction’ was defined as ≤ 6 on the Global Functioning-Social/Current Scale (GF-Social/Current; (Piskulic et al., 2011)) OR ≤ 69 on the MIRECC Global Assessment of Functioning-Social Scale (MIRECC GAF-Social; (Niv et al., 2007)). Participants above these cutoffs in the ‘functional’ range additionally had no history of psychiatric diagnoses based on the Mini-International Neuropsychiatric Interview (MINI) for DSM-5 (Sheehan et al., 1997), and were not taking psychotropic medications. Clinical ratings/assessments were performed by trained assessors.

**Recruitment.** To capture the full range of social functioning, approximately one third of the sample was recruited in the socially “functional” range (defined above), while the rest were recruited in the socially “dysfunctional” range (defined above). To ensure the sample was rich in clinical dimensions of interest, recruitment aimed for those in the “dysfunctional” range to be approximately distributed as follows: 20% with a psychotic disorder (based on via Mini International Neuropsychiatric Interview [MINI] for DSM-5; Sheehan et al., 1998), 20% with social anxiety disorder (based on via MINI-5), and 20% with autism spectrum disorder (based on Autism Diagnostic Observation Schedule 2nd Edition [ADOS-2] revised scoring; Hus & Lord, 2014; Lord et al., 2012). While recruitment was not based on diagnosis, in our analytic sample, 47%, 22%, and 11% respectively, met criteria for social anxiety, autism spectrum, and psychosis-spectrum disorders. An additional 19% met criteria for two or more of these disorders.

**Clinical Assessments and Inter-Rater Reliability.** Clinician-rated functioning measures were rated based on clinical consensus meetings supervised by a licensed clinical psychologist. Regarding the ADOS, all assessors were certified ‘research reliable’ (i.e., 80% reliability with ‘gold standard ratings’) on the ADOS by co-author Dr. Costanza Colombi PhD, a certified ADOS trainer, before administering assessments for the study. Periodic re-verification of rating reliability was performed over the course of the study under the supervision of co-author Dr. Colombi.

# 2 Gaze Perception Task

**Task Details.** Participants completed a behavioral task in which they judged whether faces were looking at them. Stimuli consisted of greyscale face images (George, 2001) of 6 actors (3 male, 3 female) that subtended a visual angle of ~20°. To approximate the multiple layers of ambiguity that individuals encounter in the real world (i.e., naturally ambiguous social cues, often judged under less-than-ideal viewing conditions), this task manipulates both the gaze signal (*x_i_*) of the stimulus and the overall signal-to-noise ratio (SNR) of faces (*k*). Gaze direction was manipulated psychophysically, spanning 9 signal strengths ranging from not looking at me (signal strength=0; eyes averted 10°) to looking at me (signal strength=1; eyes averted 0°). The task was performed across three blocks of decreasing signal to noise ratios (SNR). SNR was manipulated by adding no (high SNR), mild (medium SNR), or moderate (low SNR) Gaussian noise to stimuli. The task consisted of 324 trials (12 per signal strength x 9 signal strengths x 3 difficulties), each consisting of a 2000ms stimulus and a 1-500ms inter-stimulus interval (ISI). Responses < 200ms were removed. Sample stimuli are shown below.

**Calculating a Choice-Based Measure of Sensitivity.** To estimate a measure of sensitivity derived only from choice data (as a point of comparison for drift sensitivity measures in the main text), a logistic curve was fitted to the number of each subject’s eye contact endorsement (i.e., number of “yes” responses) across the mean-centered eye contact signal strengths. The model was used to estimate two key parameters: the threshold and slope of the function (both at 50% endorsement of eye contact). Threshold indexes one’s *self-referential bias* to perceive eye contact, where higher threshold values indicate less self-referential bias. Slope indexes one’s *sensitivity* to subtle changes in gaze direction, where higher slope values indicate greater sensitivity. Similarly to the DDMs (see Supplement 3), this model was implemented as a hierarchical Bayesian model and characterized how self-referential bias (threshold parameter) and sensitivity (slope parameter) varied across different SNR levels. The model estimated separate, correlated, slope and threshold parameters for each SNR level. The model used a non-centered parameterization and weakly informative priors: N(0,1) for mean parameters, positive-bound N(0,0.2) for variance parameters, and LKJ_correlation(1) for Cholesky factors used to estimate covariance matrices across SNR levels. Models were run using 5000 warmup and 5000 postwarmup draws per 10 chains, resulting in 50,000 postwarmup draws in cmdstanr. Several indices suggested that parameters had converged to target distributions (well-mixed chains, all Rhat < 1.01, autocorrelation ~0 by a lag of 30, no divergences). For analysis, we focused on the slope parameter as a choice-based measure of sensitivity because it most closely resembles the “drift sensitivity” parameter of the DDM. The main distinction is that choice-based sensitivity is derived only from choice data, while drift sensitivity is derived from choice and RT concurrently.

**Visualizing Basic Choice and RT Behavior.** Plots below are based on choice and RT data from the full sample. As values of gaze signals increase, it becomes increasingly clear (from the gaze direction of the stimuli) that the face in the stimulus is looking at the participant. As values of gaze signals decrease, it becomes increasingly clear (from the gaze direction of the stimuli) that the face in the stimulus is not looking at the participant.

# 3 Drift Diffusion Model (DDM)

**DDM Overview.** We used Hierarchical Bayesian Drift Diffusion Models (DDMs) (Ratcliff & McKoon, 2008) to characterize the cognitive processes driving choice and RT behavior during the self-referential gaze perception task. According to the DDM, gaze perception can be conceptualized as a process of evidence accumulation: the process of extracting and integrating information from the environment to make a decision. Each subject begins accumulating evidence from a start point, indexing initial expectations that others are looking at them. From there, how efficiently they accumulate information is determined by their drift rate. Higher magnitude drift rates mean more efficiency and, in turn, faster and more accurate/consistent responses. The threshold separation controls how much evidence is needed before deciding, indexing caution in trading speed for accuracy. Finally, non-decision time (NDT) captures time for encoding and motor execution. Together, these key parameters determine choice and RT behavior on any given trial (i) for a given subject (j). The time at which the accumulated evidence first hits a boundary (yielding a RT) and the boundary that is hit (yielding a choice) are modeled as a joint outcome of a Wiener diffusion process that is governed by four parameters: (1) trial-level drift rate (determined by subject-level drift sensitivity, subject-level drift bias, and trial-level gaze signal), (2) subject-level threshold separation, (3) subject-level start point, and (4) subject-level NDT. As such, the DDM parameters account for information about RT and choices simultaneously.

**Model Specification.** The present study adapted the DDM used by Lasagna et al. (2026) to account for the fact the present task manipulated both the gaze signal (*x_i_*) of the stimulus and the signal-to-noise ratio (SNR) of faces (*k*). The gaze signal (x_i_) varied pseudorandomly from trial-to-trial and was modeled as a continuous predictor of the trial-level drift rate. Namely, trial-level drift rates ($\delta_{i}$) were determined by: 1) how strong the physical gaze signal is (*x_i_*; mean-centered), 2) their *drift sensitivity* ($\delta_{jk}^{sensitivity}$), a parameter capturing how evidence accumulation varied in response to physical changes in self-referential gaze signals, and 3) their *drift bias* ($\delta_{jk}^{bias}$), a parameter capturing their perceptual bias for self-referential information, regardless of the actual gaze signal. Higher drift sensitivity indicated that individuals are more attuned to subtle changes in social signals, like eye gaze. Drift bias > 0 showed a perceptual bias for direct gaze, and drift bias < 0 showed a bias for indirect gaze. The relation between drift sensitivity, drift bias, gaze signals, and trial-level drift rates were captured in Equation S1:

**Trial-level drift rate** $\boldsymbol{(}\delta_{i})=Probit(\delta_{jk}^{bias}\text{+}\delta_{jk}^{sensitivity}$ * $x_{i})*10-5$	(S1)

As part of the non-centered parameterization of the model (described further below), parameters were sampled in a “standard” space and transformed to a desired value range. Equation S1 shows that, for the trial-level drift rates, this step involved a probit transformation, which had the added benefit of allowing for a non-linear mapping between gaze signals (x_i_) and trial-level drift rates (δ_i_). Other cognitive parameters—threshold separation (response caution), start point (expectancy bias), and NDT (motor/sensory processes)—were assumed to be trait-level processes that did not vary in response to trial-by-trial changes in gaze signals. However, as discussed below, we did assume that several of these other cognitive parameters were likely to be influenced by decreases in SNR level across blocks.

The SNR level (*k*) was manipulated categorically across three blocks, where block 1 was high SNR (no noise), block 2 was medium SNR (low noise), and block 3 was low SNR (high noise). The categorical effect of SNR on the parameters was modeled by estimating the drift sensitivity, drift bias, threshold separation, and start point as separate, correlated parameters across the three SNR levels (*k*)*.* NDT was fixed across conditions to help simplify the models as it was not of primary interest.

As mentioned previously, the model used a non-centered parameterization, which can aid sampling for complex models. This means that parameters are sampled in a “standardized” space and are later transformed and scaled to their desired range of values. Models also used weakly informative priors: N(0,1) for mean parameters, positive-bound N(0,0.2) for variance parameters, and LKJ_correlation(1) for the Cholesky factor used to estimate the covariance matrices across SNR levels.

**Testing and Implementation.** Models were run in cmdstanr on a high-performance computing cluster using 5,000 warmup samples, 5,000 post-warmup draws per chain, and 10 chains, resulting in 50,000 postwarmup draws. We assessed convergence by ensuring trace plots showed well-mixed chains, Rhat values were < 1.01 for all parameters, autocorrelation was ~0 by a lag of 30, and there were no divergences. All indices suggested that parameters had converged to target distributions. Our previous work showed that this model could recover parameters well and generally made accurate predictions (Lasagna et al. 2026). As such, we did not repeat those modeling steps here.

**Model comparisons.** To ensure that our primary model–that modeled effects of SNR level on parameters–best accounted for performance, we compared this model to a simpler “baseline” model that did not include the effects of SNR level parameters. Results shown below indicate that it was necessary to account for the effects of SNR on parameters to fully account for performance on the task.

| **Model** | **ELPD** | **Delta ELPD (SE)** |
| --- | --- | --- |
| Full (SNR Effects) | -23,532.65 | -- (--) |
| Baseline (No SNR Effects) | -24,687.64 | -1154.99 (47.24) |

*Note*: Models are presented in order from best- to worst-fitting based on the expected log pointwise predictive density (ELPD) within each group. Higher ELPD indicates better model fit. Differences in out-of-sample predictive accuracy were assessed relative to the best fitting model based on changes in the ELPD (Delta ELPD). Uncertainty around ∆ELPD-LOO is captured through the standard error (SE) of the estimated pointwise ELPD differences. An absolute change in ELPD < 1 SE is weak evidence for improved predictive accuracy.

# ANALYSIS SUPPLEMENT

# 4 Preliminary Analyses

## 4.1 SNR Effects

Our first preliminary analysis looked at how SNR levels impacted the parameters of the DDM. Repeated measures Bayesian ANOVA (using anovaBF in BayesFactor package) indicated that–as SNR decreased–participants became less cautious (i.e., lower threshold separation values), had less sensitivity to gaze signals (i.e., lower drift sensitivity values), and showed more self-referential perceptual biases (i.e., higher drift bias values). SNR did not appear to impact expectancy biases (start point values).

## 4.2 Correlations Between Parameters Across SNR Levels

Our second preliminary analyses examined how highly correlated our parameter of interest (drift sensitivity) was across the different SNR levels. Even though drift sensitivity values were impacted by SNR on the task, correlations between drift sensitivity values across the different SNR levels indicated that these values were HIGHLY correlated across conditions. In other words, these parameters likely reflected slight variants of the same underlying process (Sripada & Weigard, 2021; Stevenson et al., 2024; Weigard & Sripada, 2021). Therefore, for the purposes of simplifying analyses, we averaged drift sensitivity parameters across SNR levels for analyses.

## 4.3 Correlations Between Principal Components for Different Clinical Dimensions

## 4.4 Distributions of Key Variables

*Note:* Values for social functioning, social cognition, psychosis, social anxiety, and autism principal components (PC) are in arbitrary units.

## 4.5 Distributions of Clinical Variables by Diagnostic Group

*Note:* Values for psychosis, social anxiety, and autism principal components (PC) are in arbitrary units.

# 5 Primary Analyses

Below are complete outputs for regression analyses reported in the main text. In these regressions, we predicted symptoms (psychosis proneness, social anxiety, autism traits) and functioning (social cognition, social functioning) from a null (intercept-age-and-sex only model) at Step 1. At Step 2, we added drift sensitivity to evaluate whether sensitivity of evidence accumulation to gaze cues was predictive of these symptom and functional domains. Each model was run in brms using standardized variables. Variance parameters were given weakly informative priors (positive bound student_t(3, 0, 2.5)). Moderately informative priors were used for predictors (Normal(0,0.5)), informed by the fact that associations between social cognition, symptoms, and functioning in prior work often have small-to-medium-sized effects. Regressions were run using 4 parallel chains, with 1000 warmup samples and 5000 post-warmup samples per chain, resulting in 20,000 post-warmup samples.

## 5.1 Predicting Social Cognition (Controlling for Sex, Age)

| **Model** | **ELPD** | **ΔELPD (SE)** | **BF** | **R^2^ (ΔR^2^)** | **Predictor** | **BF** | **M [95% HDI]** | **Frequentist Version** |
| --- | --- | --- | --- | --- | --- | --- | --- | --- |
| 1 | -156.27 | -- (--) | -- (--/--) | 0.024 (--) |  |  |  |  |
|  |  |  |  |  | Intercept | 0.166 | -0.01 [-0.34, 0.32] | β=0.04, t=0.23, p=0.818 |
|  |  |  |  |  | Sex | 0.468 | 0.12 [-0.25, 0.48] | β=0.12, t=0.64, p=0.526 |
|  |  |  |  |  | Age | 0.187 | -0.02 [-0.2, 0.16] | β=0.02, t=0.23, p=0.816 |
| 2 | -152.29 | 3.98 (3.65) | 36.77 | 0.106 (0.082) |  |  |  |  |
|  |  |  |  |  | Intercept | 0.191 | 0.08 [-0.25, 0.43] | β=0.16, t=0.8, p=0.424 |
|  |  |  |  |  | Sex | 0.397 | -0.03 [-0.41, 0.35] | β=-0.05, t=-0.22, p=0.827 |
|  |  |  |  |  | Age | 0.229 | -0.07 [-0.24, 0.11] | β=-0.01, t=-0.12, p=0.902 |
|  |  |  |  |  | Drift Sensitivity | 68.777 | 0.31 [0.13, 0.5]* | β=0.25, t=2.2, p=0.030* |

## 5.2 Predicting Social Functioning (Controlling for Sex, Age)

| **Model** | **ELPD** | **ΔELPD (SE)** | **BF** | **R^2^ (ΔR^2^)** | **Predictor** | **BF** | **M [95% HDI]** | **Frequentist Version** |
| --- | --- | --- | --- | --- | --- | --- | --- | --- |
| 1 | -161.17 | -- (--) | -- (--/--) | 0.032 (--) |  |  |  |  |
|  |  |  |  |  | Intercept | 0.257 | 0.14 [-0.23, 0.51] | β=0.19, t=0.71, p=0.477 |
|  |  |  |  |  | Sex | 0.674 | -0.2 [-0.61, 0.21] | β=-0.28, t=-0.93, p=0.354 |
|  |  |  |  |  | Age | 0.194 | -0.03 [-0.21, 0.17] | β=-0.05, t=-0.47, p=0.641 |
| 2 | -158.79 | 2.38 (2.32) | 3.75 | 0.074 (0.042) |  |  |  |  |
|  |  |  |  |  | Intercept | 0.388 | 0.22 [-0.15, 0.57] | β=0.27, t=1.21, p=0.230 |
|  |  |  |  |  | Sex | 1.173 | -0.3 [-0.71, 0.1] | β=-0.37, t=-1.5, p=0.137 |
|  |  |  |  |  | Age | 0.197 | -0.04 [-0.23, 0.14] | β=-0.05, t=-0.53, p=0.599 |
|  |  |  |  |  | Drift Sensitivity | 3.805 | 0.23 [0.04, 0.42]* | β=0.26, t=2.68, p=0.009* |

### 5.2.1 Predicting Social Functioning (Controlling for Sex, Age, Social Cognition)

| **Model** | **ELPD** | **ΔELPD (SE)** | **BF** | **R^2^ (ΔR^2^)** | **Predictor** | **BF** | **M [95% HDI]** | **Frequentist Version** |
| --- | --- | --- | --- | --- | --- | --- | --- | --- |
| 1 | -161.17 | -- (--) | -- (--/--) | 0.032 (--) |  |  |  |  |
|  |  |  |  |  | Intercept | 0.257 | 0.14 [-0.23, 0.51] | β=0.19, t=0.71, p=0.477 |
|  |  |  |  |  | Sex | 0.674 | -0.2 [-0.61, 0.21] | β=-0.28, t=-0.93, p=0.354 |
|  |  |  |  |  | Age | 0.194 | -0.03 [-0.21, 0.17] | β=-0.05, t=-0.47, p=0.641 |
| 2 | -161.26 | -0.09 (1.32) | 0.46 | 0.05 (0.018) |  |  |  |  |
|  |  |  |  |  | Intercept | 0.249 | 0.15 [-0.22, 0.52] | β=0.2, t=0.76, p=0.451 |
|  |  |  |  |  | Sex | 0.696 | -0.21 [-0.63, 0.2] | β=-0.28, t=-0.97, p=0.337 |
|  |  |  |  |  | Age | 0.195 | -0.02 [-0.21, 0.17] | β=-0.03, t=-0.3, p=0.768 |
|  |  |  |  |  | Social Cognition PC | 0.457 | 0.13 [-0.06, 0.32] | β=0.13, t=1.25, p=0.214 |
| 3 | -159.59 | 1.58 (2.4) | 0.87 | 0.08 (0.048) |  |  |  |  |
|  |  |  |  |  | Intercept | 0.383 | 0.22 [-0.14, 0.59] | β=0.27, t=1.21, p=0.230 |
|  |  |  |  |  | Sex | 1.155 | -0.3 [-0.7, 0.12] | β=-0.37, t=-1.49, p=0.139 |
|  |  |  |  |  | Age | 0.19 | -0.03 [-0.21, 0.16] | β=-0.04, t=-0.43, p=0.666 |
|  |  |  |  |  | Social Cognition PC | 0.235 | 0.05 [-0.15, 0.25] | β=0.05, t=0.51, p=0.614 |
|  |  |  |  |  | Drift Sensitivity | 1.731 | 0.22 [0.02, 0.42]* | β=0.24, t=2.44, p=0.016* |

## 5.3 Predicting Psychosis (Controlling for Sex, Age)

| **Model** | **ELPD** | **ΔELPD (SE)** | **BF** | **R^2^ (ΔR^2^)** | **Predictor** | **BF** | **M [95% HDI]** | **Frequentist Version** |
| --- | --- | --- | --- | --- | --- | --- | --- | --- |
| 1 | -153.31 | -- (--) | -- (--/--) | 0.04 (--) |  |  |  |  |
|  |  |  |  |  | Intercept | 0.198 | -0.07 [-0.45, 0.31] | β=-0.13, t=-0.62, p=0.538 |
|  |  |  |  |  | Sex | 0.427 | 0.06 [-0.35, 0.48] | β=0.08, t=0.34, p=0.733 |
|  |  |  |  |  | Age | 0.485 | -0.13 [-0.32, 0.07] | β=-0.12, t=-1.31, p=0.192 |
| 2 | -151.49 | 1.82 (2.33) | 2.71 | 0.082 (0.042) |  |  |  |  |
|  |  |  |  |  | Intercept | 0.254 | -0.14 [-0.5, 0.23] | β=-0.23, t=-1.18, p=0.24 |
|  |  |  |  |  | Sex | 0.547 | 0.16 [-0.25, 0.57] | β=0.2, t=0.92, p=0.359 |
|  |  |  |  |  | Age | 0.481 | -0.13 [-0.32, 0.05] | β=-0.12, t=-1.37, p=0.174 |
|  |  |  |  |  | Drift Sensitivity | 2.624 | -0.23 [-0.41, -0.02]* | β=-0.24, t=-2.3, p=0.024* |

## 5.4 Predicting Social Anxiety (Controlling for Sex, Age)

| **Model** | **ELPD** | **ΔELPD (SE)** | **BF** | **R^2^ (ΔR^2^)** | **Predictor** | **BF** | **M [95% HDI]** | **Frequentist Version** |
| --- | --- | --- | --- | --- | --- | --- | --- | --- |
| 1 | -152.18 | -- (--) | -- (--/--) | 0.053 (--) |  |  |  |  |
|  |  |  |  |  | Intercept | 0.541 | -0.28 [-0.64, 0.1] | β=-0.35, t=-1.99, p=0.05 |
|  |  |  |  |  | Sex | 2.039 | 0.37 [-0.05, 0.78] | β=0.49, t=2.18, p=0.031* |
|  |  |  |  |  | Age | 0.318 | -0.09 [-0.28, 0.1] | β=-0.1, t=-1.16, p=0.248 |
| 2 | -150.28 | 1.9 (2.14) | 2.58 | 0.09 (0.037) |  |  |  |  |
|  |  |  |  |  | Intercept | 1.138 | -0.36 [-0.71, 0.01] | β=-0.44, t=-2.65, p=0.009* |
|  |  |  |  |  | Sex | 5.443 | 0.48 [0.06, 0.88]* | β=0.61, t=2.93, p=0.004* |
|  |  |  |  |  | Age | 0.318 | -0.09 [-0.28, 0.09] | β=-0.1, t=-1.22, p=0.226 |
|  |  |  |  |  | Drift Sensitivity | 2.285 | -0.22 [-0.41, -0.03]* | β=-0.25, t=-2.61, p=0.010* |

## 5.5 Predicting Autism Traits (Controlling for Sex, Age)

| **Model** | **ELPD** | **ΔELPD (SE)** | **BF** | **R^2^ (ΔR^2^)** | **Predictor** | **BF** | **M [95% HDI]** | **Frequentist Version** |
| --- | --- | --- | --- | --- | --- | --- | --- | --- |
| 1 | -161.3 | -- (--) | -- (--/--) | 0.025 (--) |  |  |  |  |
|  |  |  |  |  | Intercept | 0.191 | 0.06 [-0.3, 0.42] | β=0.06, t=0.26, p=0.797 |
|  |  |  |  |  | Sex | 0.462 | -0.1 [-0.51, 0.29] | β=-0.11, t=-0.47, p=0.639 |
|  |  |  |  |  | Age | 0.189 | -0.01 [-0.2, 0.18] | β=-0.02, t=-0.22, p=0.825 |
| 2 | -160.93 | 0.37 (2.04) | 0.88 | 0.056 (0.031) |  |  |  |  |
|  |  |  |  |  | Intercept | 0.19 | 0 [-0.35, 0.38] | β=-0.02, t=-0.11, p=0.914 |
|  |  |  |  |  | Sex | 0.436 | -0.02 [-0.42, 0.41] | β=-0.01, t=-0.04, p=0.972 |
|  |  |  |  |  | Age | 0.195 | 0.01 [-0.19, 0.19] | β=0, t=0, p=0.998 |
|  |  |  |  |  | Drift Sensitivity | 0.912 | -0.17 [-0.37, 0.01] | β=-0.18, t=-1.39, p=0.168 |

# 6 Supplemental Analyses

## 6.1 Controlling for IQ

Below are the complete outputs for the regression supplemental analyses referenced in the main text. The goal here was to re-run the previous regressions reported above and evaluate whether the results remained after also controlling for IQ as an added control variable. IQ was measured using the full-scale IQ (FSIQ2) scores from the Wechsler Abbreviated Scale of Intelligence—Second Edition (Wechsler, 2018). Each regression was re-run using the same specifications as those detailed above.

### 6.1.1 Predicting Social Cognition (Controlling for Sex, Age, IQ)

| **Model** | **ELPD** | **ΔELPD (SE)** | **BF** | **R^2^ (ΔR^2^)** | **Predictor** | **BF** | **M [95% HDI]** | **Frequentist Version** |
| --- | --- | --- | --- | --- | --- | --- | --- | --- |
| 1 | -156.59 | -- (--) | -- (--/--) | 0.039 (--) |  |  |  |  |
|  |  |  |  |  | Intercept | 0.175 | -0.02 [-0.36, 0.31] | β=-0.01, t=-0.03, p=0.975 |
|  |  |  |  |  | Sex | 0.549 | 0.16 [-0.21, 0.53] | β=0.18, t=0.92, p=0.360 |
|  |  |  |  |  | Age | 0.187 | 0.01 [-0.18, 0.18] | β=0.05, t=0.62, p=0.534 |
|  |  |  |  |  | IQ | 0.385 | 0.11 [-0.07, 0.31] | β=0.18, t=1.55, p=0.124 |
| 2 | -153.63 | 2.96 (3.63) | 20.12 | 0.109 (0.07) |  |  |  |  |
|  |  |  |  |  | Intercept | 0.195 | 0.08 [-0.27, 0.43] | β=0.11, t=0.52, p=0.607 |
|  |  |  |  |  | Sex | 0.395 | -0.01 [-0.4, 0.38] | β=0.03, t=0.11, p=0.912 |
|  |  |  |  |  | Age | 0.226 | -0.06 [-0.25, 0.12] | β=0.02, t=0.2, p=0.841 |
|  |  |  |  |  | IQ | 0.205 | 0.04 [-0.15, 0.23] | β=0.13, t=1.09, p=0.277 |
|  |  |  |  |  | Drift Sensitivity | 24.332 | 0.3 [0.11, 0.49]* | β=0.22, t=1.94, p=0.055 |

### 6.1.2 Predicting Social Functioning (Controlling for Sex, Age, IQ)

| **Model** | **ELPD** | **ΔELPD (SE)** | **BF** | **R^2^ (ΔR^2^)** | **Predictor** | **BF** | **M [95% HDI]** | **Frequentist Version** |
| --- | --- | --- | --- | --- | --- | --- | --- | --- |
| 1 | -162.03 | -- (--) | -- (--/--) | 0.042 (--) |  |  |  |  |
|  |  |  |  |  | Intercept | 0.245 | 0.12 [-0.24, 0.51] | β=0.17, t=0.63, p=0.528 |
|  |  |  |  |  | Sex | 0.57 | -0.17 [-0.57, 0.26] | β=-0.25, t=-0.82, p=0.415 |
|  |  |  |  |  | IQ | 0.248 | 0.07 [-0.14, 0.25] | β=-0.03, t=-0.33, p=0.745 |
|  |  |  |  |  | Age | 0.21 | -0.02 [-0.21, 0.18] | β=0.07, t=0.61, p=0.545 |
| 2 | -159.8 | 2.23 (2.38) | 3.28 | 0.081 (0.04) |  |  |  |  |
|  |  |  |  |  | Intercept | 0.333 | 0.21 [-0.16, 0.58] | β=0.26, t=1.13, p=0.259 |
|  |  |  |  |  | Sex | 0.982 | -0.28 [-0.7, 0.14] | β=-0.35, t=-1.38, p=0.171 |
|  |  |  |  |  | IQ | 0.22 | 0.05 [-0.13, 0.24] | β=-0.04, t=-0.46, p=0.646 |
|  |  |  |  |  | Age | 0.211 | -0.03 [-0.22, 0.15] | β=0.03, t=0.34, p=0.734 |
|  |  |  |  |  | Drift Sensitivity | 3.719 | 0.23 [0.03, 0.41]* | β=0.26, t=2.58, p=0.011* |

#### 6.1.2.1 Follow-Up Predicting Social Functioning (Controlling for Sex, IQ, Social Cognition)

| **Model** | **ELPD** | **ΔELPD (SE)** | **BF** | **R^2^ (ΔR^2^)** | **Predictor** | **BF** | **M [95% HDI]** | **Frequentist Version** |
| --- | --- | --- | --- | --- | --- | --- | --- | --- |
| 1 | -162.11 | -- (--) | -- (--/--) | 0.058 (--) |  |  |  |  |
|  |  |  |  |  | Intercept | 0.243 | 0.14 [-0.24, 0.49] | β=0.18, t=0.66, p=0.508 |
|  |  |  |  |  | Sex | 0.633 | -0.19 [-0.59, 0.24] | β=-0.25, t=-0.84, p=0.403 |
|  |  |  |  |  | Age | 0.192 | -0.01 [-0.2, 0.19] | β=-0.02, t=-0.2, p=0.842 |
|  |  |  |  |  | Social Cognition PC | 0.436 | 0.12 [-0.07, 0.31] | β=0.06, t=0.52, p=0.605 |
|  |  |  |  |  | IQ | 0.238 | 0.06 [-0.13, 0.26] | β=0.13, t=1.17, p=0.246 |
| 2 | -160.62 | 1.49 (1.97) | 1.71 | 0.087 (0.029) |  |  |  |  |
|  |  |  |  |  | Intercept | 0.346 | 0.21 [-0.17, 0.59] | β=0.26, t=1.13, p=0.262 |
|  |  |  |  |  | Sex | 0.994 | -0.28 [-0.71, 0.14] | β=-0.35, t=-1.36, p=0.176 |
|  |  |  |  |  | Age | 0.206 | -0.03 [-0.21, 0.16] | β=-0.04, t=-0.37, p=0.710 |
|  |  |  |  |  | Social Cognition PC | 0.234 | 0.05 [-0.15, 0.25] | β=0.03, t=0.35, p=0.729 |
|  |  |  |  |  | IQ | 0.208 | 0.05 [-0.14, 0.24] | β=0.05, t=0.5, p=0.616 |
|  |  |  |  |  | Drift Sensitivity | 1.847 | 0.21 [0.01, 0.41]* | β=0.24, t=2.38, p=0.019* |

### 6.1.3 Predicting Psychosis (Controlling for Sex, Age, IQ)

| **Model** | **ELPD** | **ΔELPD (SE)** | **BF** | **R^2^ (ΔR^2^)** | **Predictor** | **BF** | **M [95% HDI]** | **Frequentist Version** |
| --- | --- | --- | --- | --- | --- | --- | --- | --- |
| 1 | -153.66 | -- (--) | -- (--/--) | 0.057 (--) |  |  |  |  |
|  |  |  |  |  | Intercept | 0.195 | -0.02 [-0.39, 0.34] | β=-0.04, t=-0.19, p=0.853 |
|  |  |  |  |  | Sex | 0.426 | 0 [-0.44, 0.41] | β=-0.02, t=-0.08, p=0.934 |
|  |  |  |  |  | IQ | 0.413 | -0.12 [-0.32, 0.08] | β=-0.14, t=-1.53, p=0.128 |
|  |  |  |  |  | Age | 0.58 | -0.14 [-0.34, 0.04] | β=-0.14, t=-1.13, p=0.262 |
| 2 | -152.11 | 1.55 (2.3) | 2.29 | 0.094 (0.037) |  |  |  |  |
|  |  |  |  |  | Intercept | 0.213 | -0.1 [-0.47, 0.28] | β=-0.15, t=-0.7, p=0.488 |
|  |  |  |  |  | Sex | 0.472 | 0.1 [-0.31, 0.54] | β=0.1, t=0.41, p=0.684 |
|  |  |  |  |  | IQ | 0.352 | -0.11 [-0.3, 0.09] | β=-0.14, t=-1.59, p=0.115 |
|  |  |  |  |  | Age | 0.568 | -0.14 [-0.33, 0.04] | β=-0.13, t=-1.01, p=0.314 |
|  |  |  |  |  | Drift Sensitivity | 2.073 | -0.22 [-0.41, -0.02]* | β=-0.24, t=-2.25, p=0.027* |

### 6.1.4 Predicting Social Anxiety (Controlling for Sex, Age, IQ)

| **Model** | **ELPD** | **ΔELPD (SE)** | **BF** | **R^2^ (ΔR^2^)** | **Predictor** | **BF** | **M [95% HDI]** | **Frequentist Version** |
| --- | --- | --- | --- | --- | --- | --- | --- | --- |
| 1 | -152.09 | -- (--) | -- (--/--) | 0.072 (--) |  |  |  |  |
|  |  |  |  |  | Intercept | 0.406 | -0.23 [-0.62, 0.14] | β=-0.29, t=-1.63, p=0.106 |
|  |  |  |  |  | Sex | 1.286 | 0.32 [-0.1, 0.74] | β=0.42, t=1.83, p=0.07 |
|  |  |  |  |  | IQ | 0.535 | -0.14 [-0.33, 0.05] | β=-0.11, t=-1.36, p=0.175 |
|  |  |  |  |  | Age | 0.373 | -0.11 [-0.3, 0.08] | β=-0.14, t=-1.58, p=0.118 |
| 2 | -150.44 | 1.65 (2.15) | 2.1 | 0.105 (0.033) |  |  |  |  |
|  |  |  |  |  | Intercept | 0.743 | -0.32 [-0.68, 0.05] | β=-0.4, t=-2.35, p=0.021* |
|  |  |  |  |  | Sex | 3.039 | 0.42 [0.01, 0.84]* | β=0.54, t=2.62, p=0.01* |
|  |  |  |  |  | IQ | 0.42 | -0.12 [-0.3, 0.08] | β=-0.11, t=-1.4, p=0.163 |
|  |  |  |  |  | Age | 0.357 | -0.11 [-0.29, 0.08] | β=-0.12, t=-1.3, p=0.198 |
|  |  |  |  |  | Drift Sensitivity | 1.998 | -0.21 [-0.39, -0.01]* | β=-0.24, t=-2.33, p=0.022* |

### 6.1.5 Predicting Autism Traits (Controlling for Sex, Age, IQ)

| **Model** | **ELPD** | **ΔELPD (SE)** | **BF** | **R^2^ (ΔR^2^)** | **Predictor** | **BF** | **M [95% HDI]** | **Frequentist Version** |
| --- | --- | --- | --- | --- | --- | --- | --- | --- |
| 1 | -162.35 | -- (--) | -- (--/--) | 0.033 (--) |  |  |  |  |
|  |  |  |  |  | Intercept | 0.192 | 0.05 [-0.32, 0.41] | β=0.05, t=0.24, p=0.814 |
|  |  |  |  |  | Sex | 0.44 | -0.08 [-0.49, 0.34] | β=-0.1, t=-0.44, p=0.661 |
|  |  |  |  |  | IQ | 0.215 | 0.04 [-0.16, 0.23] | β=-0.02, t=-0.16, p=0.874 |
|  |  |  |  |  | Age | 0.2 | 0 [-0.19, 0.19] | β=0.03, t=0.21, p=0.831 |
| 2 | -161.88 | 0.47 (2.04) | 0.9 | 0.062 (0.029) |  |  |  |  |
|  |  |  |  |  | Intercept | 0.184 | -0.02 [-0.4, 0.34] | β=-0.04, t=-0.17, p=0.867 |
|  |  |  |  |  | Sex | 0.432 | 0 [-0.4, 0.44] | β=0.01, t=0.04, p=0.970 |
|  |  |  |  |  | IQ | 0.217 | 0.05 [-0.15, 0.24] | β=0.01, t=0.06, p=0.949 |
|  |  |  |  |  | Age | 0.195 | 0.01 [-0.18, 0.2] | β=0.04, t=0.34, p=0.737 |
|  |  |  |  |  | Drift Sensitivity | 0.927 | -0.17 [-0.36, 0.03] | β=-0.18, t=-1.42, p=0.158 |

## 6.2 Traditional Metrics (RT and Choice-Based Sensitivity)

Below are the outputs for the supplemental regressions referenced in the main text. The goal was to re-run the previous regressions reported above and evaluate whether credible results were also observed when using “traditional” measures of gaze task performance (RT and choice-based sensitivity) as predictors. Each regression model was re-run using the same specifications as those detailed above in the previous section.

### 6.2.1 Predicting Social Cognition (Using RT and Choice-Based Sensitivity Metrics)

| **Model** | **ELPD** | **ΔELPD (SE)** | **BF** | **R^2^ (ΔR^2^)** | **Predictor** | **BF** | **M [95% HDI]** | **Frequentist Version** |
| --- | --- | --- | --- | --- | --- | --- | --- | --- |
| 1 | -156.27 | – (–) | – (–/–) | 0.024 (–) |  |  |  |  |
|  |  |  |  |  | Intercept | 0.166 | -0.01 [-0.34, 0.32] | β=0.04, t=0.23, p=0.818 |
|  |  |  |  |  | Sex | 0.468 | 0.12 [-0.25, 0.48] | β=0.12, t=0.64, p=0.526 |
|  |  |  |  |  | Age | 0.187 | -0.02 [-0.2, 0.16] | β=0.02, t=0.23, p=0.816 |
| 2 | -156.75 | -0.48 (2.65) | 0.67 | 0.052 (0.028) |  |  |  |  |
|  |  |  |  |  | Intercept | 0.193 | 0.06 [-0.29, 0.39] | β=0.13, t=0.71, p=0.476 |
|  |  |  |  |  | Sex | 0.401 | 0.05 [-0.31, 0.43] | β=0, t=0.01, p=0.995 |
|  |  |  |  |  | Age | 0.181 | -0.03 [-0.21, 0.14] | β=-0.02, t=-0.21, p=0.831 |
|  |  |  |  |  | Sensitivity | 0.709 | 0.16 [-0.06, 0.37] | β=0.25, t=1.81, p=0.074 |
| 3 | -157.19 | -0.92 (0.32) | 0.18 | 0.029 (0.006) |  |  |  |  |
|  |  |  |  |  | Intercept | 0.183 | -0.02 [-0.35, 0.32] | β=0.03, t=0.16, p=0.875 |
|  |  |  |  |  | Sex | 0.474 | 0.13 [-0.24, 0.5] | β=0.14, t=0.7, p=0.486 |
|  |  |  |  |  | Age | 0.197 | -0.02 [-0.2, 0.16] | β=0.03, t=0.32, p=0.746 |
|  |  |  |  |  | RT | 0.175 | 0.02 [-0.14, 0.2] | β=0.03, t=0.39, p=0.695 |

### 6.2.2 Predicting Social Functioning (Using RT and Choice-Based Sensitivity Metrics)

| **Model** | **ELPD** | **ΔELPD (SE)** | **BF** | **R^2^ (ΔR^2^)** | **Predictor** | **BF** | **M [95% HDI]** | **Frequentist Version** |
| --- | --- | --- | --- | --- | --- | --- | --- | --- |
| 1 | -161.17 | – (–) | – (–/–) | 0.032 (–) |  |  |  |  |
|  |  |  |  |  | Intercept | 0.257 | 0.14 [-0.23, 0.51] | β=0.19, t=0.71, p=0.477 |
|  |  |  |  |  | Sex | 0.674 | -0.2 [-0.61, 0.21] | β=-0.28, t=-0.93, p=0.354 |
|  |  |  |  |  | Age | 0.194 | -0.03 [-0.21, 0.17] | β=-0.05, t=-0.47, p=0.641 |
| 2 | -162.35 | -1.19 (0.56) | 0.2 | 0.038 (0.006) |  |  |  |  |
|  |  |  |  |  | Intercept | 0.246 | 0.15 [-0.22, 0.51] | β=0.19, t=0.7, p=0.486 |
|  |  |  |  |  | Sex | 0.68 | -0.2 [-0.61, 0.21] | β=-0.28, t=-0.91, p=0.366 |
|  |  |  |  |  | Age | 0.208 | -0.03 [-0.22, 0.16] | β=-0.05, t=-0.47, p=0.64 |
|  |  |  |  |  | Sensitivity | 0.199 | 0 [-0.2, 0.19] | β=0, t=0.02, p=0.984 |
| 3 | -161.26 | -0.09 (1.39) | 0.47 | 0.05 (0.018) |  |  |  |  |
|  |  |  |  |  | Intercept | 0.276 | 0.17 [-0.2, 0.52] | β=0.21, t=0.84, p=0.405 |
|  |  |  |  |  | Sex | 0.767 | -0.23 [-0.64, 0.16] | β=-0.31, t=-1.09, p=0.276 |
|  |  |  |  |  | Age | 0.226 | -0.05 [-0.24, 0.14] | β=-0.06, t=-0.65, p=0.514 |
|  |  |  |  |  | RT | 0.469 | -0.13 [-0.32, 0.07] | β=-0.15, t=-1.34, p=0.184 |

### 6.2.3 Predicting Psychosis (Using RT and Choice-Based Sensitivity Metrics)

| **Model** | **ELPD** | **ΔELPD (SE)** | **BF** | **R^2^ (ΔR^2^)** | **Predictor** | **BF** | **M [95% HDI]** | **Frequentist Version** |
| --- | --- | --- | --- | --- | --- | --- | --- | --- |
| 1 | -153.31 | – (–) | – (–/–) | 0.04 (–) |  |  |  |  |
|  |  |  |  |  | Intercept | 0.198 | -0.07 [-0.45, 0.31] | β=-0.13, t=-0.62, p=0.538 |
|  |  |  |  |  | Sex | 0.427 | 0.06 [-0.35, 0.48] | β=0.08, t=0.34, p=0.733 |
|  |  |  |  |  | Age | 0.485 | -0.13 [-0.32, 0.07] | β=-0.12, t=-1.31, p=0.192 |
| 2 | -153.2 | 0.11 (1.07) | 0.44 | 0.057 (0.017) |  |  |  |  |
|  |  |  |  |  | Intercept | 0.202 | -0.08 [-0.44, 0.3] | β=-0.14, t=-0.69, p=0.495 |
|  |  |  |  |  | Sex | 0.441 | 0.07 [-0.34, 0.48] | β=0.09, t=0.41, p=0.684 |
|  |  |  |  |  | Age | 0.369 | -0.11 [-0.31, 0.07] | β=-0.1, t=-1.12, p=0.266 |
|  |  |  |  |  | Sensitivity | 0.447 | -0.12 [-0.32, 0.06] | β=-0.12, t=-1.49, p=0.14 |
| 3 | -152.64 | 0.67 (1.81) | 0.93 | 0.067 (0.027) |  |  |  |  |
|  |  |  |  |  | Intercept | 0.228 | -0.11 [-0.48, 0.26] | β=-0.18, t=-0.95, p=0.342 |
|  |  |  |  |  | Sex | 0.47 | 0.11 [-0.3, 0.53] | β=0.14, t=0.66, p=0.512 |
|  |  |  |  |  | Age | 0.323 | -0.1 [-0.29, 0.09] | β=-0.09, t=-1.02, p=0.31 |
|  |  |  |  |  | RT | 0.934 | 0.17 [-0.02, 0.37] | β=0.2, t=2.02, p=0.046* |

### 6.2.4 Predicting Social Anxiety (Using RT and Choice-Based Sensitivity Metrics)

| **Model** | **ELPD** | **ΔELPD (SE)** | **BF** | **R^2^ (ΔR^2^)** | **Predictor** | **BF** | **M [95% HDI]** | **Frequentist Version** |
| --- | --- | --- | --- | --- | --- | --- | --- | --- |
| 1 | -152.18 | – (–) | – (–/–) | 0.053 (–) |  |  |  |  |
|  |  |  |  |  | Intercept | 0.541 | -0.28 [-0.64, 0.1] | β=-0.35, t=-1.99, p=0.05* |
|  |  |  |  |  | Sex | 2.039 | 0.37 [-0.05, 0.78] | β=0.49, t=2.18, p=0.031* |
|  |  |  |  |  | Age | 0.318 | -0.09 [-0.28, 0.1] | β=-0.1, t=-1.16, p=0.248 |
| 2 | -152.56 | -0.38 (0.98) | 0.34 | 0.066 (0.014) |  |  |  |  |
|  |  |  |  |  | Intercept | 0.636 | -0.29 [-0.67, 0.07] | β=-0.36, t=-2.05, p=0.043* |
|  |  |  |  |  | Sex | 2.3 | 0.39 [-0.02, 0.81] | β=0.5, t=2.26, p=0.026* |
|  |  |  |  |  | Age | 0.264 | -0.08 [-0.26, 0.12] | β=-0.08, t=-0.94, p=0.351 |
|  |  |  |  |  | Sensitivity | 0.354 | -0.1 [-0.3, 0.09] | β=-0.12, t=-1.28, p=0.205 |
| 3 | -152.27 | -0.1 (1.34) | 0.46 | 0.069 (0.017) |  |  |  |  |
|  |  |  |  |  | Intercept | 0.704 | -0.31 [-0.68, 0.06] | β=-0.38, t=-2.3, p=0.023* |
|  |  |  |  |  | Sex | 2.631 | 0.41 [-0.01, 0.83] | β=0.54, t=2.51, p=0.014* |
|  |  |  |  |  | Age | 0.271 | -0.07 [-0.26, 0.12] | β=-0.08, t=-0.96, p=0.342 |
|  |  |  |  |  | RT | 0.441 | 0.13 [-0.07, 0.32] | β=0.15, t=1.4, p=0.165 |

### 6.2.5 Predicting Autism Traits (Using RT and Choice-Based Sensitivity Metrics)

| **Model** | **ELPD** | **ΔELPD (SE)** | **BF** | **R^2^ (ΔR^2^)** | **Predictor** | **BF** | **M [95% HDI]** | **Frequentist Version** |
| --- | --- | --- | --- | --- | --- | --- | --- | --- |
| 1 | -161.3 | – (–) | – (–/–) | 0.025 (–) |  |  |  |  |
|  |  |  |  |  | Intercept | 0.191 | 0.06 [-0.3, 0.42] | β=0.06, t=0.26, p=0.797 |
|  |  |  |  |  | Sex | 0.462 | -0.1 [-0.51, 0.29] | β=-0.11, t=-0.47, p=0.639 |
|  |  |  |  |  | Age | 0.189 | -0.01 [-0.2, 0.18] | β=-0.02, t=-0.22, p=0.825 |
| 2 | -162.6 | -1.3 (0.84) | 0.26 | 0.036 (0.012) |  |  |  |  |
|  |  |  |  |  | Intercept | 0.198 | 0.07 [-0.28, 0.43] | β=0.08, t=0.38, p=0.703 |
|  |  |  |  |  | Sex | 0.488 | -0.11 [-0.52, 0.28] | β=-0.15, t=-0.6, p=0.551 |
|  |  |  |  |  | Age | 0.209 | -0.02 [-0.21, 0.17] | β=-0.04, t=-0.38, p=0.706 |
|  |  |  |  |  | Sensitivity | 0.273 | 0.07 [-0.13, 0.27] | β=0.09, t=0.59, p=0.554 |
| 3 | -161.87 | -0.57 (0.93) | 0.31 | 0.039 (0.015) |  |  |  |  |
|  |  |  |  |  | Intercept | 0.194 | 0.04 [-0.33, 0.4] | β=0.03, t=0.16, p=0.877 |
|  |  |  |  |  | Sex | 0.463 | -0.07 [-0.49, 0.34] | β=-0.08, t=-0.33, p=0.742 |
|  |  |  |  |  | Age | 0.193 | 0.01 [-0.18, 0.2] | β=0, t=-0.04, p=0.968 |
|  |  |  |  |  | RT | 0.328 | 0.09 [-0.1, 0.28] | β=0.09, t=0.95, p=0.342 |

## 6.3 Expectancy Bias (DDM Start Point)

Below are the outputs for the supplemental regressions referenced in the main text. The goal was to re-run the previous regressions reported above and evaluate whether credible results were also observed when using parameters that indexed initial expectancy biases (i.e., start point) as predictors. Each regression model was re-run using the same specifications as those detailed above in the previous section.

### 6.3.1 Predicting Social Cognition (Using DDM Start Point)

| **Model** | **ELPD** | **ΔELPD (SE)** | **BF** | **R^2^ (ΔR^2^)** | **Predictor** | **BF** | **M [95% HDI]** | **Frequentist Version** |
| --- | --- | --- | --- | --- | --- | --- | --- | --- |
| 1 | -156.27 | – (–) | – (–/–) | 0.024 (–) |  |  |  |  |
|  |  |  |  |  | Intercept | 0.166 | -0.01 [-0.34, 0.32] | β=0.04, t=0.23, p=0.818 |
|  |  |  |  |  | Sex | 0.468 | 0.12 [-0.25, 0.48] | β=0.12, t=0.64, p=0.526 |
|  |  |  |  |  | Age | 0.187 | -0.02 [-0.2, 0.16] | β=0.02, t=0.23, p=0.816 |
| 2 | -157.16 | -0.9 (0.54) | 0.2 | 0.031 (0.007) |  |  |  |  |
|  |  |  |  |  | Intercept | 0.167 | -0.01 [-0.34, 0.32] | β=0.04, t=0.23, p=0.818 |
|  |  |  |  |  | Sex | 0.474 | 0.12 [-0.24, 0.49] | β=0.12, t=0.66, p=0.513 |
|  |  |  |  |  | Age | 0.185 | -0.02 [-0.2, 0.16] | β=0.02, t=0.28, p=0.783 |
|  |  |  |  |  | Start Point | 0.213 | 0.04 [-0.13, 0.22] | β=0.07, t=0.75, p=0.453 |

### 6.3.2 Predicting Social Functioning (Using DDM Start Point)

| **Model** | **ELPD** | **ΔELPD (SE)** | **BF** | **R^2^ (ΔR^2^)** | **Predictor** | **BF** | **M [95% HDI]** | **Frequentist Version** |
| --- | --- | --- | --- | --- | --- | --- | --- | --- |
| 1 | -161.17 | – (–) | – (–/–) | 0.032 (–) |  |  |  |  |
|  |  |  |  |  | Intercept | 0.257 | 0.14 [-0.23, 0.51] | β=0.19, t=0.71, p=0.477 |
|  |  |  |  |  | Sex | 0.674 | -0.2 [-0.61, 0.21] | β=-0.28, t=-0.93, p=0.354 |
|  |  |  |  |  | Age | 0.194 | -0.03 [-0.21, 0.17] | β=-0.05, t=-0.47, p=0.641 |
| 2 | -161.88 | -0.71 (0.59) | 0.23 | 0.04 (0.009) |  |  |  |  |
|  |  |  |  |  | Intercept | 0.249 | 0.14 [-0.23, 0.49] | β=0.19, t=0.7, p=0.487 |
|  |  |  |  |  | Sex | 0.668 | -0.2 [-0.6, 0.2] | β=-0.28, t=-0.92, p=0.36 |
|  |  |  |  |  | Age | 0.204 | -0.03 [-0.22, 0.16] | β=-0.04, t=-0.43, p=0.669 |
|  |  |  |  |  | Start Point | 0.233 | 0.06 [-0.13, 0.25] | β=0.05, t=0.53, p=0.599 |

### 6.3.3 Predicting Psychosis (Using DDM Start Point)

| **Model** | **ELPD** | **ΔELPD (SE)** | **BF** | **R^2^ (ΔR^2^)** | **Predictor** | **BF** | **M [95% HDI]** | **Frequentist Version** |
| --- | --- | --- | --- | --- | --- | --- | --- | --- |
| 1 | -153.31 | – (–) | – (–/–) | 0.04 (–) |  |  |  |  |
|  |  |  |  |  | Intercept | 0.198 | -0.07 [-0.45, 0.31] | β=-0.13, t=-0.62, p=0.538 |
|  |  |  |  |  | Sex | 0.427 | 0.06 [-0.35, 0.48] | β=0.08, t=0.34, p=0.733 |
|  |  |  |  |  | Age | 0.485 | -0.13 [-0.32, 0.07] | β=-0.12, t=-1.31, p=0.192 |
| 2 | -154.41 | -1.1 (0.21) | 0.2 | 0.047 (0.006) |  |  |  |  |
|  |  |  |  |  | Intercept | 0.196 | -0.07 [-0.42, 0.32] | β=-0.13, t=-0.61, p=0.546 |
|  |  |  |  |  | Sex | 0.44 | 0.06 [-0.37, 0.46] | β=0.08, t=0.35, p=0.731 |
|  |  |  |  |  | Age | 0.475 | -0.13 [-0.33, 0.06] | β=-0.12, t=-1.33, p=0.185 |
|  |  |  |  |  | Start Point | 0.203 | -0.02 [-0.22, 0.17] | β=-0.02, t=-0.14, p=0.889 |

### 6.3.4 Predicting Social Anxiety (Using DDM Start Point)

| **Model** | **ELPD** | **ΔELPD (SE)** | **BF** | **R^2^ (ΔR^2^)** | **Predictor** | **BF** | **M [95% HDI]** | **Frequentist Version** |
| --- | --- | --- | --- | --- | --- | --- | --- | --- |
| 1 | -152.18 | – (–) | – (–/–) | 0.053 (–) |  |  |  |  |
|  |  |  |  |  | Intercept | 0.541 | -0.28 [-0.64, 0.1] | β=-0.35, t=-1.99, p=0.05 |
|  |  |  |  |  | Sex | 2.039 | 0.37 [-0.05, 0.78] | β=0.49, t=2.18, p=0.031* |
|  |  |  |  |  | Age | 0.318 | -0.09 [-0.28, 0.1] | β=-0.1, t=-1.16, p=0.248 |
| 2 | -153.32 | -1.14 (0.16) | 0.2 | 0.058 (0.006) |  |  |  |  |
|  |  |  |  |  | Intercept | 0.565 | -0.28 [-0.63, 0.1] | β=-0.35, t=-2, p=0.048* |
|  |  |  |  |  | Sex | 1.866 | 0.38 [-0.03, 0.79] | β=0.49, t=2.2, p=0.03* |
|  |  |  |  |  | Age | 0.307 | -0.09 [-0.28, 0.1] | β=-0.1, t=-1.14, p=0.257 |
|  |  |  |  |  | Start Point | 0.201 | 0 [-0.2, 0.19] | β=0.01, t=0.1, p=0.917 |

### 6.3.5 Predicting Autism Traits (Using DDM Start Point)

| **Model** | **ELPD** | **ΔELPD (SE)** | **BF** | **R^2^ (ΔR^2^)** | **Predictor** | **BF** | **M [95% HDI]** | **Frequentist Version** |
| --- | --- | --- | --- | --- | --- | --- | --- | --- |
| 1 | -161.3 | – (–) | – (–/–) | 0.025 (–) |  |  |  |  |
|  |  |  |  |  | Intercept | 0.191 | 0.06 [-0.3, 0.42] | β=0.06, t=0.26, p=0.797 |
|  |  |  |  |  | Sex | 0.462 | -0.1 [-0.51, 0.29] | β=-0.11, t=-0.47, p=0.639 |
|  |  |  |  |  | Age | 0.189 | -0.01 [-0.2, 0.18] | β=-0.02, t=-0.22, p=0.825 |
| 2 | -162.17 | -0.87 (0.16) | 0.19 | 0.031 (0.007) |  |  |  |  |
|  |  |  |  |  | Intercept | 0.2 | 0.06 [-0.29, 0.44] | β=0.06, t=0.26, p=0.797 |
|  |  |  |  |  | Sex | 0.477 | -0.1 [-0.52, 0.29] | β=-0.11, t=-0.47, p=0.638 |
|  |  |  |  |  | Age | 0.202 | -0.01 [-0.2, 0.18] | β=-0.02, t=-0.24, p=0.813 |
|  |  |  |  |  | Start Point | 0.194 | -0.01 [-0.2, 0.18] | β=-0.02, t=-0.23, p=0.818 |

## 6.4 Processing Bias (DDM Drift Bias)

Below are the outputs for the supplemental regressions referenced in the main text. The goal was to re-run the previous regressions reported above and evaluate whether credible results were also observed when using parameters that indexed processing biases (i.e., drift bias) as predictors. Each regression model was re-run using the same specifications as those detailed above in the previous section.

### 6.4.1 Predicting Social Cognition (Using DDM Drift Bias)

| **Model** | **ELPD** | **ΔELPD (SE)** | **BF** | **R^2^ (ΔR^2^)** | **Predictor** | **BF** | **M [95% HDI]** | **Frequentist Version** |
| --- | --- | --- | --- | --- | --- | --- | --- | --- |
| 1 | -156.27 | – (–) | – (–/–) | 0.024 (–) |  |  |  |  |
|  |  |  |  |  | Intercept | 0.166 | -0.01 [-0.34, 0.32] | β=0.04, t=0.23, p=0.818 |
|  |  |  |  |  | Sex | 0.468 | 0.12 [-0.25, 0.48] | β=0.12, t=0.64, p=0.526 |
|  |  |  |  |  | Age | 0.187 | -0.02 [-0.2, 0.16] | β=0.02, t=0.23, p=0.816 |
| 2 | -157.76 | -1.49 (1.3) | 0.37 | 0.052 (0.029) |  |  |  |  |
|  |  |  |  |  | Intercept | 0.171 | -0.02 [-0.36, 0.32] | β=0.04, t=0.26, p=0.796 |
|  |  |  |  |  | Sex | 0.46 | 0.12 [-0.26, 0.49] | β=0.13, t=0.67, p=0.503 |
|  |  |  |  |  | Age | 0.221 | -0.04 [-0.24, 0.13] | β=0.03, t=0.3, p=0.764 |
|  |  |  |  |  | Drift Bias | 0.357 | -0.11 [-0.32, 0.12] | β=0.02, t=0.11, p=0.91 |

### 6.4.2 Predicting Social Functioning (Using DDM Drift Bias)

| **Model** | **ELPD** | **ΔELPD (SE)** | **BF** | **R^2^ (ΔR^2^)** | **Predictor** | **BF** | **M [95% HDI]** | **Frequentist Version** |
| --- | --- | --- | --- | --- | --- | --- | --- | --- |
| 1 | -161.17 | – (–) | – (–/–) | 0.032 (–) |  |  |  |  |
|  |  |  |  |  | Intercept | 0.257 | 0.14 [-0.23, 0.51] | β=0.19, t=0.71, p=0.477 |
|  |  |  |  |  | Sex | 0.674 | -0.2 [-0.61, 0.21] | β=-0.28, t=-0.93, p=0.354 |
|  |  |  |  |  | Age | 0.194 | -0.03 [-0.21, 0.17] | β=-0.05, t=-0.47, p=0.641 |
| 2 | -162.04 | -0.87 (0.53) | 0.23 | 0.04 (0.008) |  |  |  |  |
|  |  |  |  |  | Intercept | 0.252 | 0.15 [-0.22, 0.51] | β=0.21, t=0.8, p=0.425 |
|  |  |  |  |  | Sex | 0.708 | -0.22 [-0.62, 0.19] | β=-0.3, t=-1.03, p=0.307 |
|  |  |  |  |  | Age | 0.205 | -0.03 [-0.22, 0.15] | β=-0.05, t=-0.49, p=0.626 |
|  |  |  |  |  | Drift Bias | 0.23 | -0.05 [-0.24, 0.15] | β=-0.08, t=-0.66, p=0.508 |

### 6.4.3 Predicting Psychosis (Using DDM Drift Bias)

| **Model** | **ELPD** | **ΔELPD (SE)** | **BF** | **R^2^ (ΔR^2^)** | **Predictor** | **BF** | **M [95% HDI]** | **Frequentist Version** |
| --- | --- | --- | --- | --- | --- | --- | --- | --- |
| 1 | -153.31 | – (–) | – (–/–) | 0.04 (–) |  |  |  |  |
|  |  |  |  |  | Intercept | 0.198 | -0.07 [-0.45, 0.31] | β=-0.13, t=-0.62, p=0.538 |
|  |  |  |  |  | Sex | 0.427 | 0.06 [-0.35, 0.48] | β=0.08, t=0.34, p=0.733 |
|  |  |  |  |  | Age | 0.485 | -0.13 [-0.32, 0.07] | β=-0.12, t=-1.31, p=0.192 |
| 2 | -154.21 | -0.89 (0.71) | 0.24 | 0.049 (0.008) |  |  |  |  |
|  |  |  |  |  | Intercept | 0.208 | -0.08 [-0.45, 0.3] | β=-0.15, t=-0.69, p=0.493 |
|  |  |  |  |  | Sex | 0.434 | 0.07 [-0.36, 0.49] | β=0.1, t=0.43, p=0.668 |
|  |  |  |  |  | Age | 0.489 | -0.13 [-0.32, 0.06] | β=-0.12, t=-1.34, p=0.184 |
|  |  |  |  |  | Drift Bias | 0.249 | 0.06 [-0.14, 0.25] | β=0.07, t=0.65, p=0.52 |

### 6.4.4 Predicting Social Anxiety (Using DDM Drift Bias)

| **Model** | **ELPD** | **ΔELPD (SE)** | **BF** | **R^2^ (ΔR^2^)** | **Predictor** | **BF** | **M [95% HDI]** | **Frequentist Version** |
| --- | --- | --- | --- | --- | --- | --- | --- | --- |
| 1 | -152.18 | – (–) | – (–/–) | 0.053 (–) |  |  |  |  |
|  |  |  |  |  | Intercept | 0.541 | -0.28 [-0.64, 0.1] | β=-0.35, t=-1.99, p=0.05 |
|  |  |  |  |  | Sex | 2.039 | 0.37 [-0.05, 0.78] | β=0.49, t=2.18, p=0.031* |
|  |  |  |  |  | Age | 0.318 | -0.09 [-0.28, 0.1] | β=-0.1, t=-1.16, p=0.248 |
| 2 | -153.11 | -0.93 (0.29) | 0.2 | 0.059 (0.006) |  |  |  |  |
|  |  |  |  |  | Intercept | 0.544 | -0.27 [-0.64, 0.1] | β=-0.34, t=-1.95, p=0.054 |
|  |  |  |  |  | Sex | 1.794 | 0.37 [-0.05, 0.78] | β=0.48, t=2.12, p=0.037* |
|  |  |  |  |  | Age | 0.304 | -0.09 [-0.28, 0.1] | β=-0.1, t=-1.15, p=0.252 |
|  |  |  |  |  | Drift Bias | 0.214 | -0.02 [-0.21, 0.16] | β=-0.01, t=-0.12, p=0.908 |

### 6.4.5 Predicting Autism Traits (Using DDM Drift Bias)

| **Model** | **ELPD** | **ΔELPD (SE)** | **BF** | **R^2^ (ΔR^2^)** | **Predictor** | **BF** | **M [95% HDI]** | **Frequentist Version** |
| --- | --- | --- | --- | --- | --- | --- | --- | --- |
| 1 | -161.3 | – (–) | – (–/–) | 0.025 (–) |  |  |  |  |
|  |  |  |  |  | Intercept | 0.191 | 0.06 [-0.3, 0.42] | β=0.06, t=0.26, p=0.797 |
|  |  |  |  |  | Sex | 0.462 | -0.1 [-0.51, 0.29] | β=-0.11, t=-0.47, p=0.639 |
|  |  |  |  |  | Age | 0.189 | -0.01 [-0.2, 0.18] | β=-0.02, t=-0.22, p=0.825 |
| 2 | -161.49 | -0.2 (1.42) | 0.5 | 0.047 (0.022) |  |  |  |  |
|  |  |  |  |  | Intercept | 0.191 | 0.04 [-0.31, 0.41] | β=0.03, t=0.17, p=0.867 |
|  |  |  |  |  | Sex | 0.448 | -0.07 [-0.47, 0.34] | β=-0.08, t=-0.33, p=0.744 |
|  |  |  |  |  | Age | 0.192 | 0 [-0.19, 0.19] | β=-0.01, t=-0.16, p=0.875 |
|  |  |  |  |  | Drift Bias | 0.495 | 0.13 [-0.06, 0.32] | β=0.15, t=1.41, p=0.162 |

# 7 References

Gelman, A., & Rubin, D. B. (1992). Inference from Iterative Simulation Using Multiple Sequences. Statistical Science, 7(4), 457–472. <https://doi.org/10.1214/ss/1177011136>

George, N., Driver, J., & Dolan, R. J. (2001). Seen gaze-direction modulates fusiform activity and its coupling with other brain areas during face processing. NeuroImage, 13(6), 1102–1112.

Kingdom, F.A.A., Prins, N., 2010. Psychophysics: A practical introduction. Elsevier Academic Press, San Diego, CA, US

Lasagna, C., Pleskac, T., Weigard, A., & Tso, I. (2026). Modeling Self-Reference in Schizophrenia: General and Specific Mechanisms Underlying Decision-Making are Differentially Associated with Paranoia. PsyArXiv. https://doi.org/10.31234/osf.io/9b23k_v3

Lee, M. D., & Wagenmakers, E.-J. (2014). Bayesian Cognitive Modeling: A Practical Course. Cambridge University Press. <https://doi.org/10.1017/CBO9781139087759>

Niv, N., Cohen, A. N., Sullivan, G., & Young, A. S. (2007). The MIRECC Version of the Global Assessment of Functioning Scale: Reliability and Validity. Psychiatric Services, 58(4), 529–535. <https://doi.org/10.1176/ps.2007.58.4.529>

Piskulic, D., Addington, J., Auther, A., & Cornblatt, B. A. (2011). Using the global functioning social and role scales in a first-episode sample. Early Intervention in Psychiatry, 5(3), 219–223. <https://doi.org/10.1111/j.1751-7893.2011.00263.x>

Schütt, H. H., Harmeling, S., Macke, J. H., & Wichmann, F. A. (2016). Painfree and accurate Bayesian estimation of psychometric functions for (potentially) overdispersed data. Vision research, 122, 105-123.

Sheehan, D., Lecrubier, Y., Harnett Sheehan, K., … Dunbar, G. (1997). The validity of the Mini International Neuropsychiatric Interview (MINI) according to the SCID-P and its reliability. European Psychiatry, 12(5), 232–241. <https://doi.org/10.1016/S0924-9338(97)83297-X>

Stevenson, N., Innes, R. J., Boag, R. J., Miletić, S., Isherwood, S. J. S., Trutti, A. C., Heathcote, A., & Forstmann, B. U. (2024). Joint Modelling of Latent Cognitive Mechanisms Shared Across Decision-Making Domains. Computational Brain & Behavior, 7(1), 1–22.

Wechsler, D. (2018). Wechsler Abbreviated Scale of Intelligence—Second Edition [Dataset]. <https://doi.org/10.1037/t15171-000>
